# Supplementary material for: Poly ADP-ribosylation of SET8 leads to aberrant H4K20 methylation in mammalian nuclear genome
Source: Commun Biol. 2022 Nov 25;5:1292. doi: 10.1038/s42003-022-04241-8 (PMC9700808; doi:10.1038/s42003-022-04241-8)
Supplement: Supplementary file 2 — Supplementary Information [file 42003_2022_4241_MOESM2_ESM.pdf]

Supplementary materials

Poly ADP-ribosylation of SET8 leads to aberrant H4K20 methylation in mammalian nuclear genome

Pierre-Olivier Estève *, Sagnik Sen*, Vishnu US, Cristian Ruse[1], Hang Gyeong Chin and Sriharsa Pradhan**

New England Biolabs Inc, 240 County Road, Ipswich, MA 01938, USA

* Equal contribution to the work.

Note: “Supplementary Figure” designations are indicated on top of the figures as “Fig. S”

Fig. S1

a

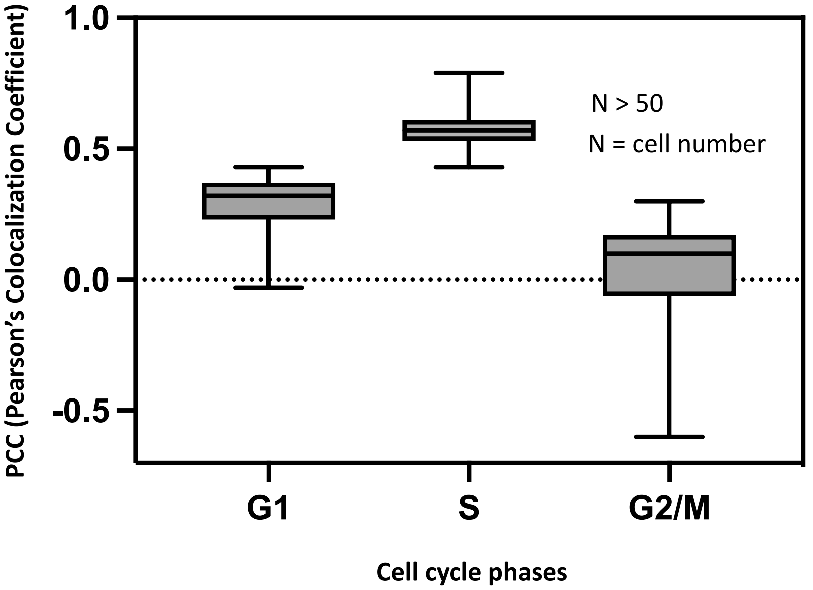

b

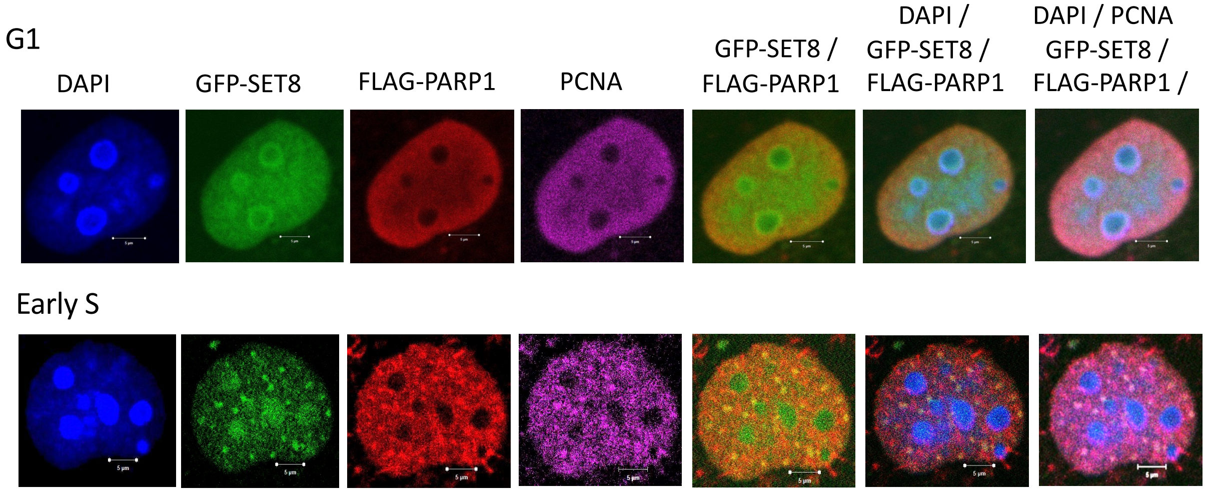

Supplementary Figure 1: Cell cycle dependent colocalization kinetics between GFP-SET8 and FLAG-PARP1. (a) Boxplot of Pearson's correlation coefficient (PCC) of cells in G1, S and G2/M. (b) Synchronized cells displaying PCNA association with SET8 and PARP1 at the onset of S-phase of the cell cycle with punctate colocalization pattern.

Fig. S2

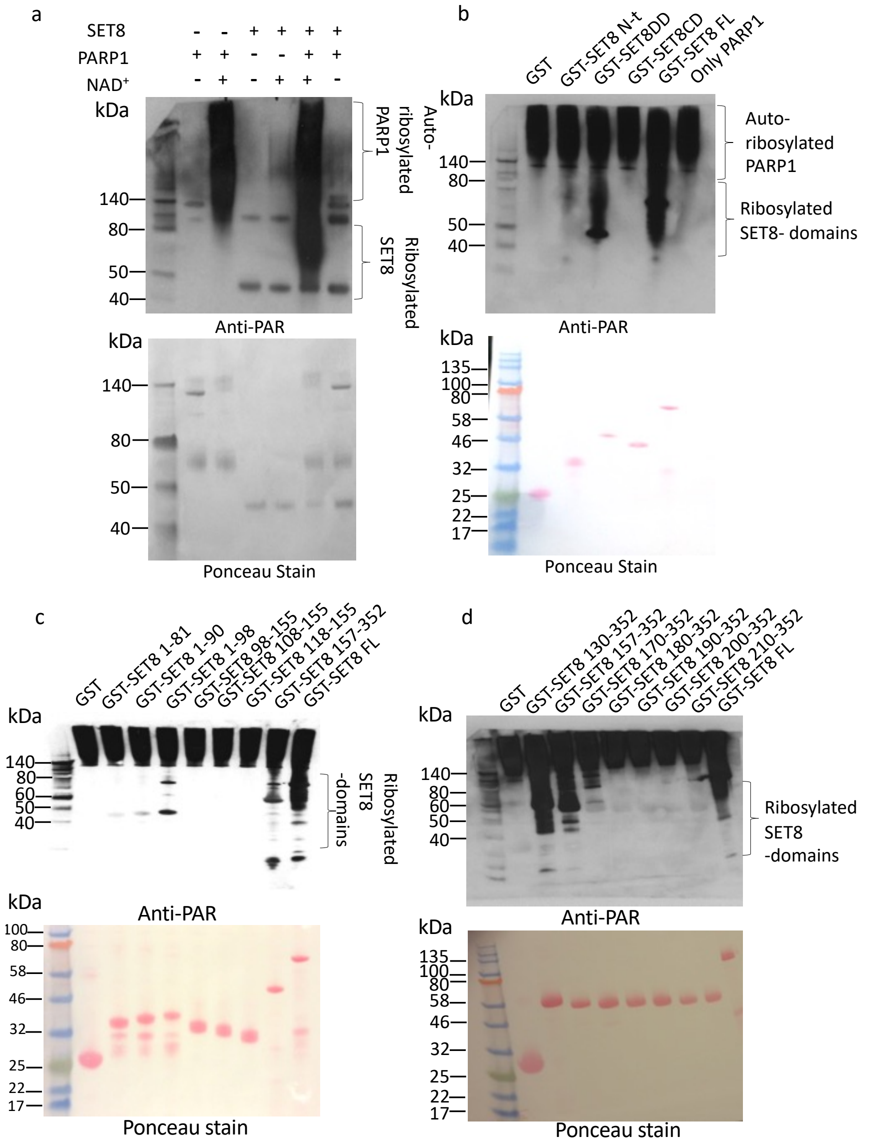

Supplementary Figure 2: Detail poly ADP-ribosylation mapping of SET8. (a) Full length SET8 is poly ADP ribosylated by PARP1. The enzymatic in vitro reaction was separated using SDS-PAGE, western blotted and probed with anti-PAR antibody. The shifted SET8 band shows the ADP-ribosylated SET8 (top). Loading of the exact blot shown using ponceau staining (bottom). (b) ADP-ribosylation of various SET8 domain in the presence of PARP1. The ribosylated SET8 GST-SET8 N-t (1-98), GST-SET8 DD (157-352) domains as well as GST-SET8 FL are shown by western blotting using anti-PAR antibody (top). Loading of the exact blot shown using ponceau staining (bottom). (c) ADP-ribosylation of various GST-SET8 regions in the presence of PARP1. GST-SET8 N-t (1-98), GST-SET8 DD (157-352) and GST-SET8 FL show ribosylation (top). Loading of the exact blot shown using ponceau staining (bottom). (d) Detailed ADP-ribosylation analysis of various truncated SET8 regions between amino acids 130-352 in the presence of PARP1. GST-SET8 (130-152), GST-SET8 (157-352), GST-SET8 (170-352) as well as GST-SET8 FL shows ADP-ribosylation (top). Loading of the exact blot shown using ponceau staining (bottom).

Fig. S3a

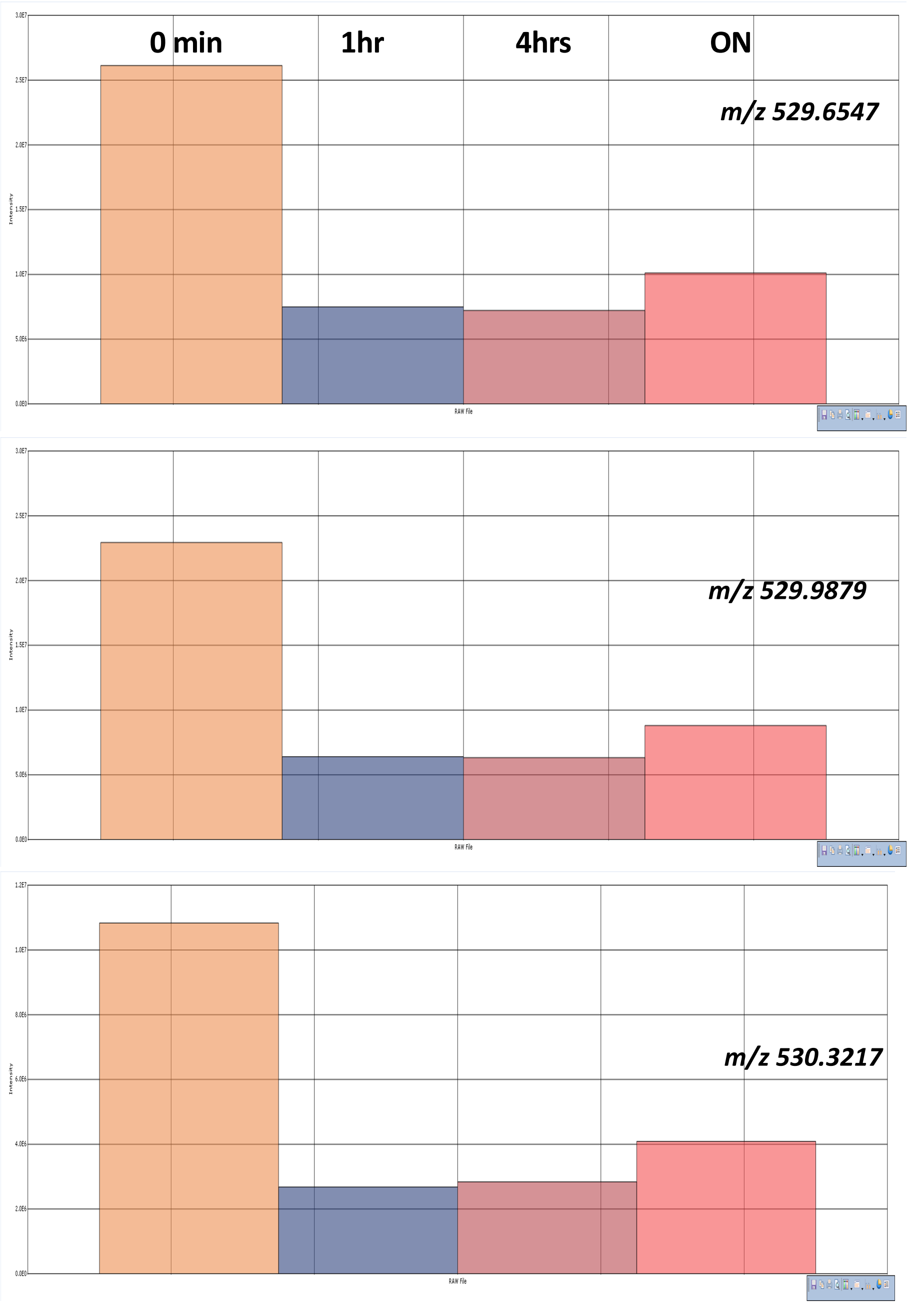

KPLAGIYRKREEK-NH2

[M+3H]3+

Fig. S3b

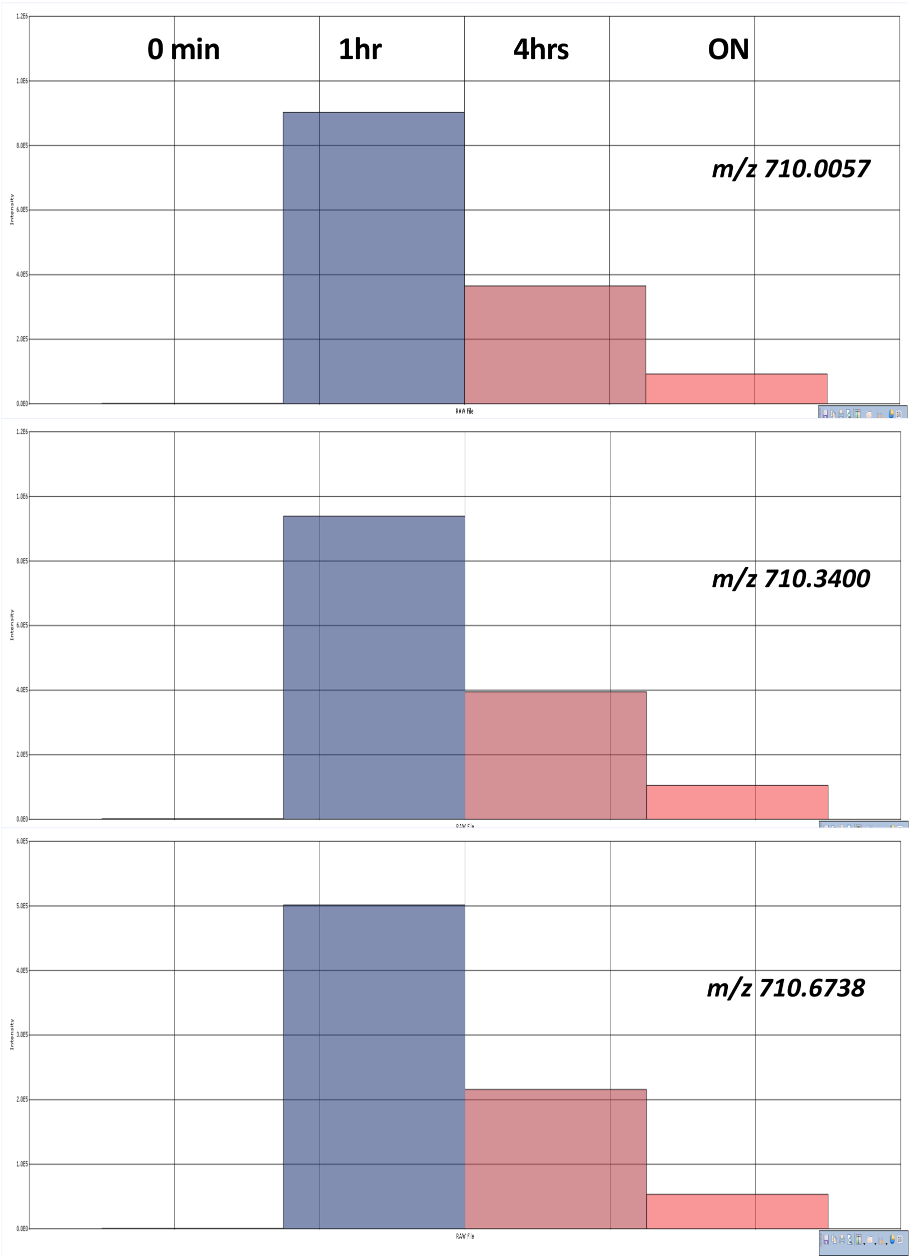

(ADPr)KPLAGIYRKREEK-NH2

[M+3H]3+

Fig. S3c

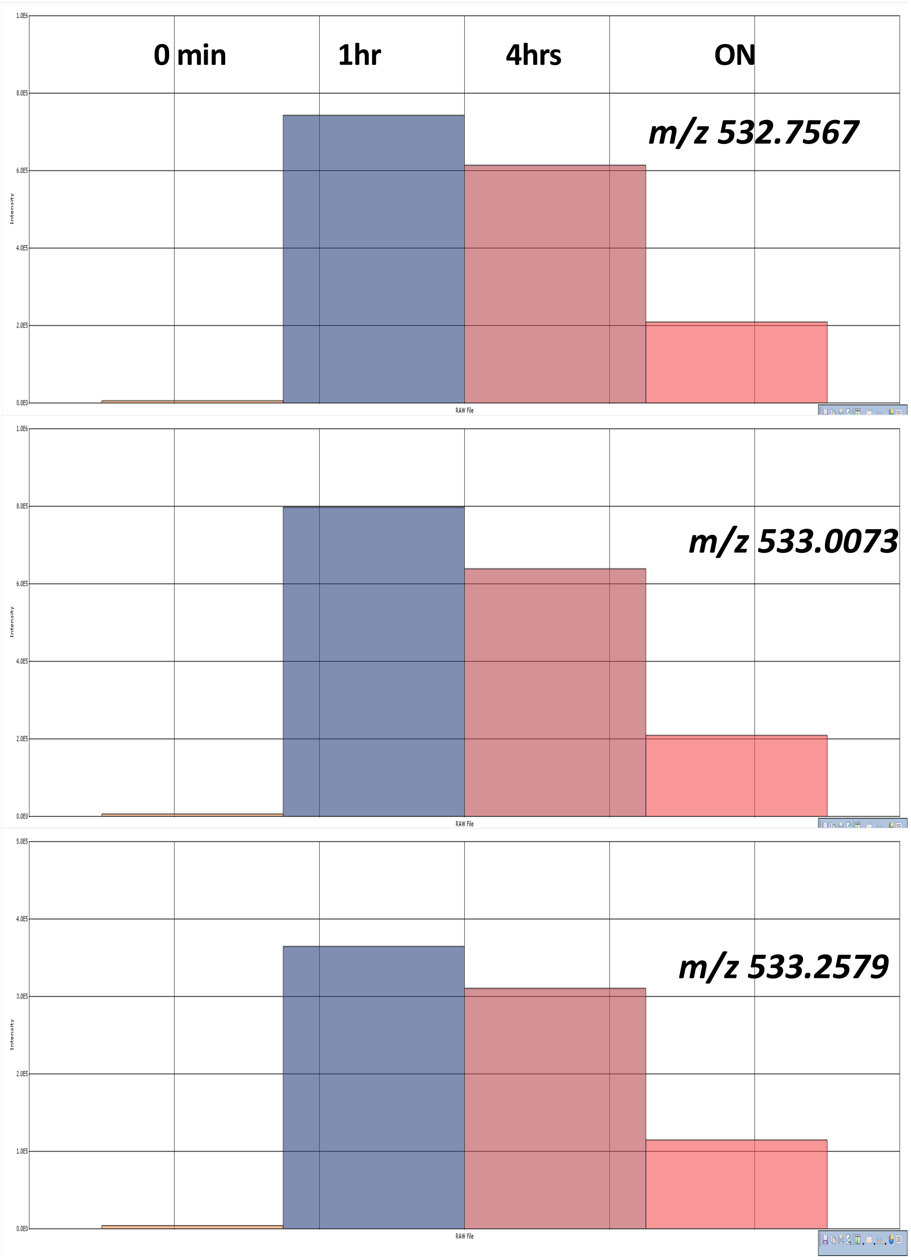

(ADPr)KPLAGIYRKREEK-NH2
[M+4H]4+

Fig. S3d

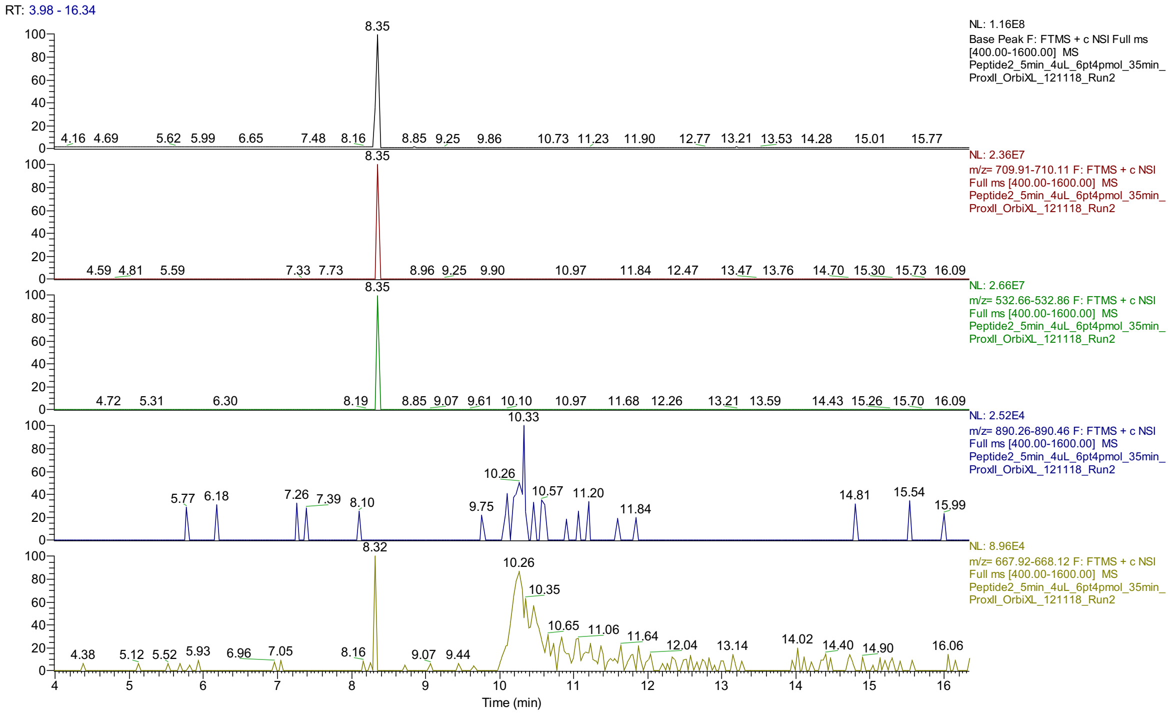

KPLAGIYRKREEK-NH2 peptide mono and di-ADP ribose conjugated forms detected at 5min reaction with PARP1

Fig. S3e

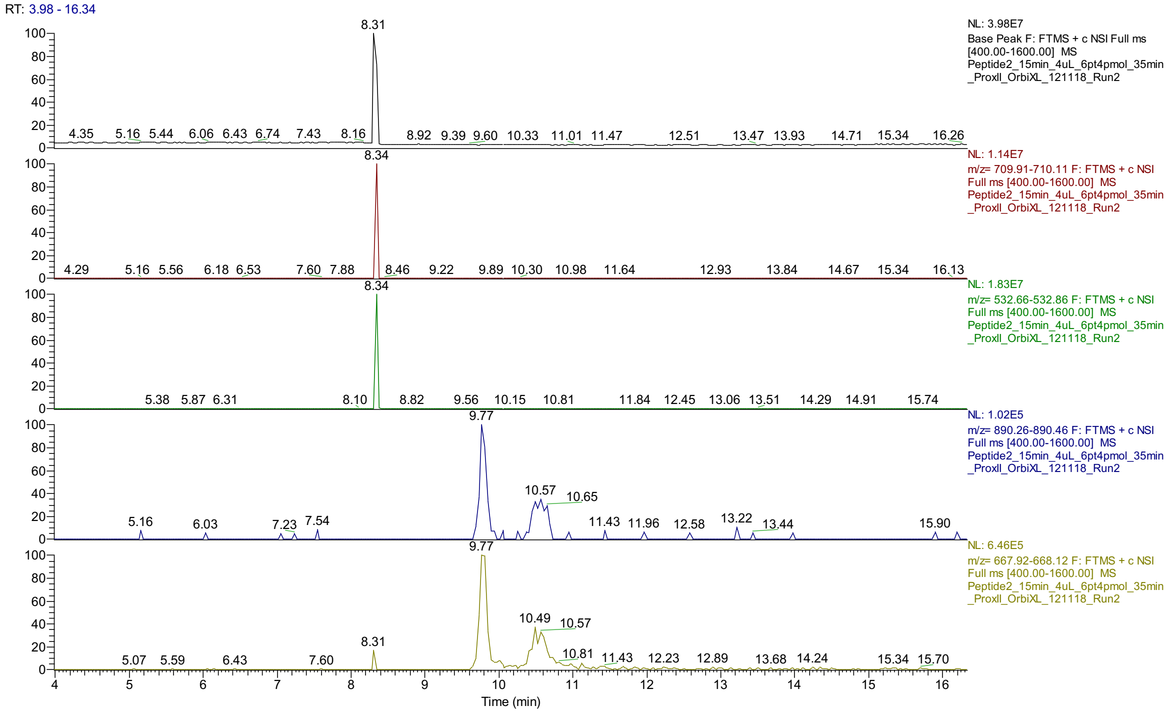

KPLAGIYRKREEK-NH2 peptide mono and diADPribo conjugated forms detected at 15min reaction with PARP1

Supplementary Figure 3: Characterization of ADP-ribosylation of SET8 synthetic peptide KPLAGIYRKREEK-NH2 using high resolution mass spectrometry and isotope quantification in nanoLC. (a) Quantification of peptide KPLAGIYRKREEK-NH2 (charge +3) at four time points: 0 min (negative control), 1hr, 4rhs and overnight (ON) reaction with PARP1. Peak areas were determined with SIEVE software package for the three main isotopes m/z 529.65449, m/z 529.98877 and m/z 530.32300, theoretical values determined with Protein Prospector MS Isotope. The figure displays the measured isotopes 0, 1 and 2 with Orbitrap MS. (b) Quantification of peptide (ADPr)KPLAGIYRKREEK-NH2 (charge +3) at four time points: 0 min (negative control), 1hr, 4rhs and overnight (ON) reaction with PARP1. Peak areas were determined with SIEVE software package for the three main isotopes m/z 710.00819, m/z 710.34247 and m/z 710.67670, theoretical values determined with Protein Prospector MS Isotope. The figure displays the measured isotopes 0, 1 and 2 with Orbitrap MS. (c) Quantification of peptide (ADPr)KPLAGIYRKREEK-NH2 (charge +4) at four time points: 0 min (negative control), 1hr, 4rhs and overnight (ON) reaction with PARP1. Peak areas were determined with SIEVE software package for the three main isotopes m/z 532.75796, m/z 533.00867 and m/z 533.25934, theoretical values determined with Protein Prospector MS Isotope. The figure displays the measured isotopes 0, 1 and 2 with Orbitrap MS. (d) Chromatographic profile of the main isotopes of (1ADPr) KPLAGIYRKREEK-NH2 and (2ADPr) KPLAGIYRKREEK-NH2 detected at 5 min reaction with PARP1. (e) Chromatographic profile of the main isotopes of (1ADPr) KPLAGIYRKREEK-NH2 and (2ADPr) KPLAGIYRKREEK-NH2 detected at 15 min reaction with PARP1.

Fig. S4a

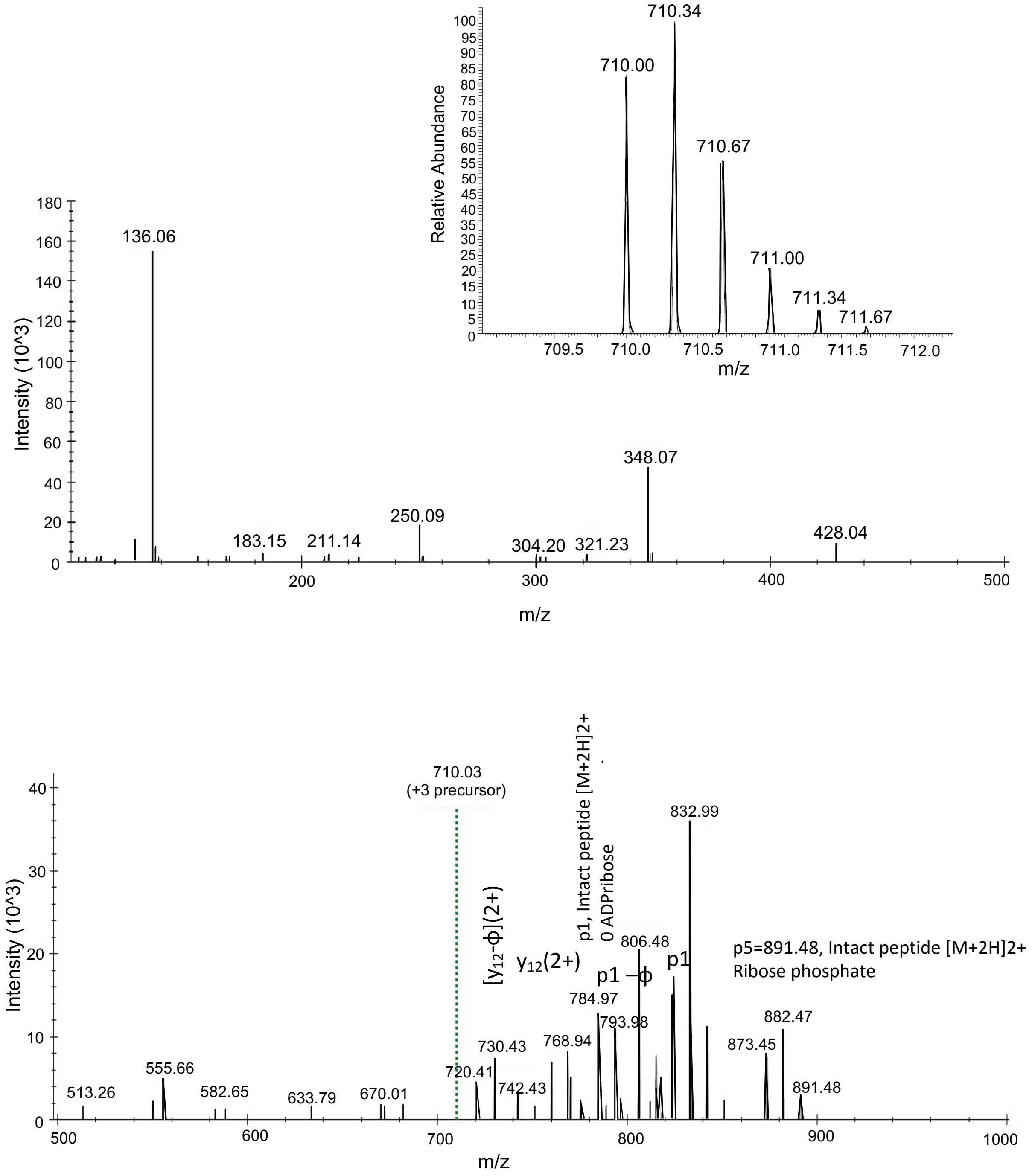

Relative Abundance
710.34
710.00
710.67
711.00
711.34
711.67
m/z
Intensity (10^3)
136.06
183.15
211.14
250.09
304.20
321.23
348.07
428.04
m/z
710.03
(+3 precursor)
832.99
p1, Intact peptide [M+2H]2+
0 ADPribose
[y12-ϕ](2+)
y12(2+)
p1 −ϕ
806.48
p1
p5=891.48, Intact peptide [M+2H]2+
Ribose phosphate
784.97
793.98
882.47
730.43
768.94
873.45
555.66
720.41
742.43
513.26
582.65
633.79
670.01
891.48
Intensity (10^3)
m/z

Fig. S4b

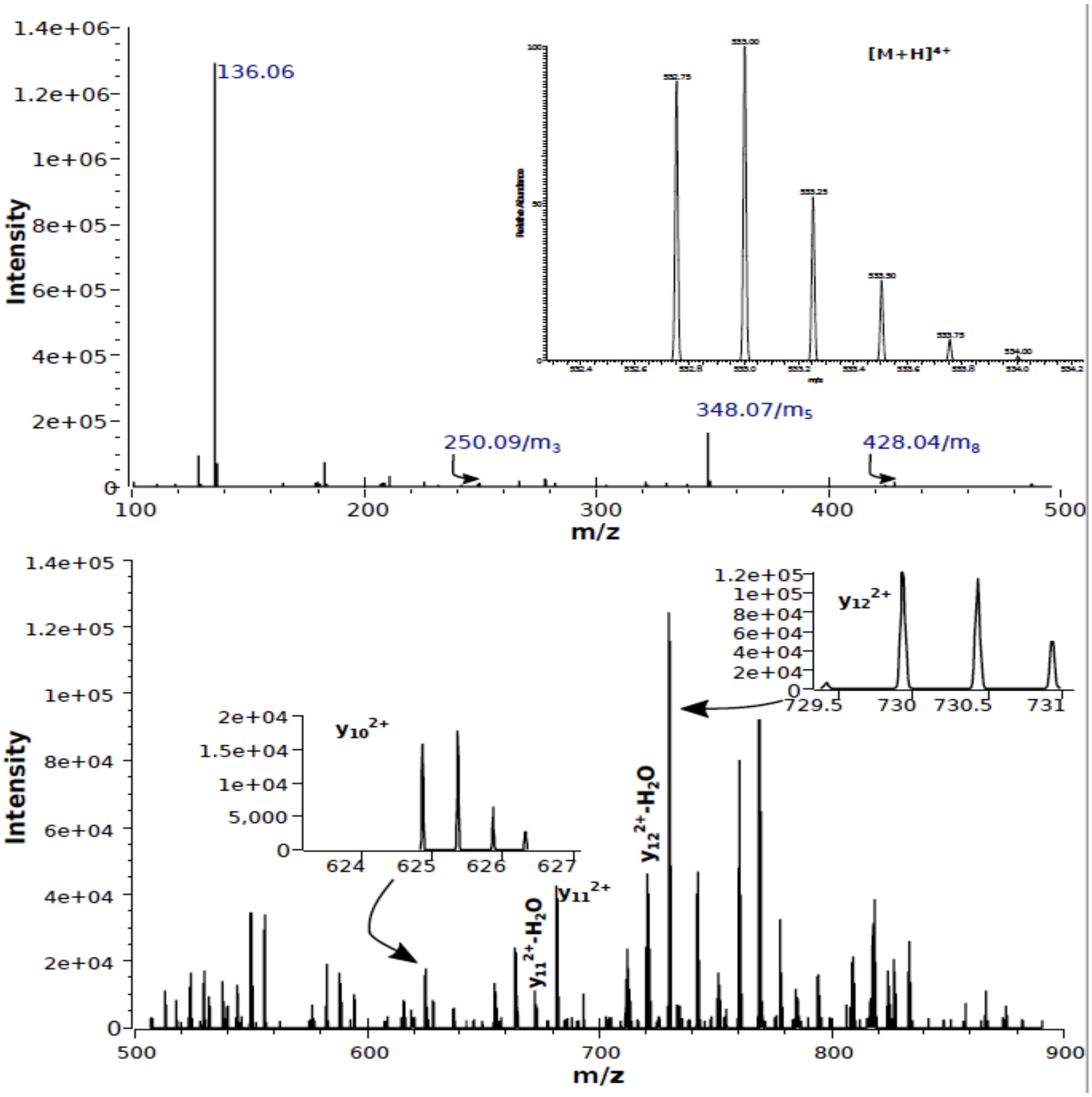

Supplementary Figure 4: Characterization of ADP-ribosylation of SET8 synthetic peptide KPLAGIYRKREEK-NH2 using high resolution/high accuracy MS/MS HCD fragmentation, [M+3H]3+ precursor m/z 710.00 and [M+4H]4+ precursor m/z 532.75. (a) Characteristics ADP-ribose modification fragmentation ions (m-ions peptide free modification fragment ions):m1= 136.1, m3=250, m5=348, m8=428 (top panel). Ion m/z 136.1 could be sourced from either tyrosine immonium ion (m/z 136.0762) or [adenine+H]+ (m/z 136.0623). Fragment ions with intact peptide p1 and intact peptide with ribose phosphate p5 are shown on the bottom panel, m/z 500 - 900. The nomenclature for ADP-ribosylation fragment ions is according to (Hengel et al, 2009). Inset shows the [M+3H]3+ precursor full scan MS spectrum. (b) Similar to charge state +3, fragmented precursor charge state +4 showed characteristics ADP-ribose modification fragment ions. Increased intensity sequencing ions are present in the bottom panel, m/z 500 - 900. Inset shows the [M+4H]4+ precursor full scan MS spectrum.

Fig. S5

(ADPr)KPLAGIYRKREEK-NH2 [M+3H]3+ m/z 710.008

	Score	*Matches*
K(ADPr)PLAGIYRKREEK	199.2	41
KPLAGIYR(ADPr)KREEK	90.5	30
KPLAGIYRK(ADPr)REEK	86.2	30
KPLAGIYRKR(ADPr)EEK	86.2	30
KPLAGIYRKRE(ADPr)EK	76.3	29
KPLAGIYRKREE(ADPr)K	113	33
KPLAGIYRKREEK(ADPr)	117	34

(ADPr)KPLAGIYRKREEK-NH2 [M+4H]4+ m/z 532.75

	Score	*Matches*
K(ADPr)PLAGIYRKREEK	159.6	37
KPLAGIYR(ADPr)KREEK	48.3	20
KPLAGIYRK(ADPr)REEK	60.4	22
KPLAGIYRKR(ADPr)EEK	53	21
KPLAGIYRKRE(ADPr)EK	60.3	23
KPLAGIYRKREE(ADPr)K	82.6	27
KPLAGIYRKREEK(ADPr)	109.6	31

Supplementary Figure 5: Characterization of ADP-ribosylation of SET8 synthetic peptide KPLAGIYRKREEK-NH2 using MS/MS CID fragmentation. [M+3H]3+ precursor m/z 710.008 and [M+4H]4+ precursor m/z 532.75 are shown. Scoring of ion propensity matches of the ADPr unit relative to seven possible amino acid locations in CID MS/MS spectra of (ADPr)KPLAGIYRKREEK-NH2.

Fig. S6a

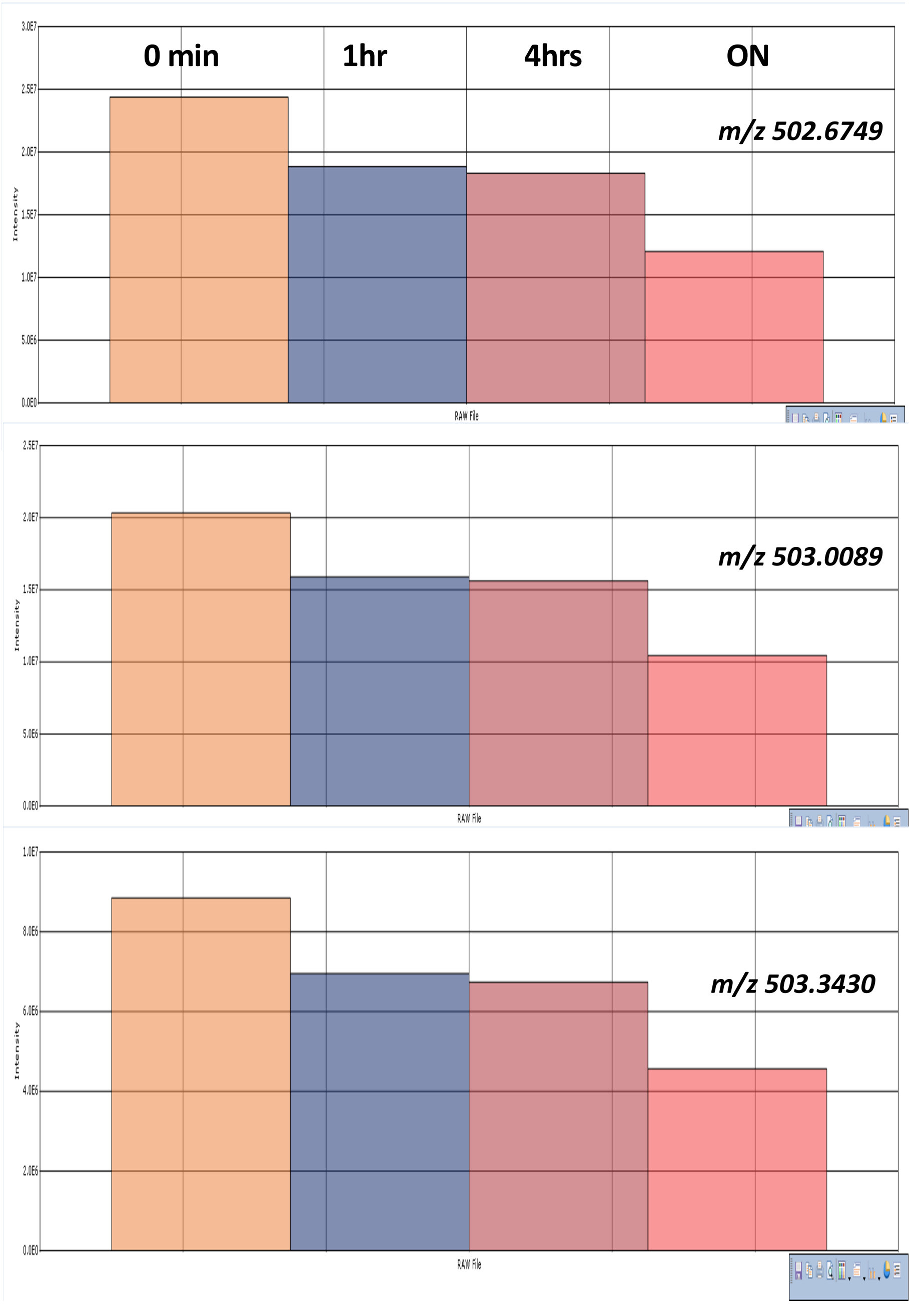

KKPIKGKQAPRKK-NH2

[M+3H]3+

Fig. S6b

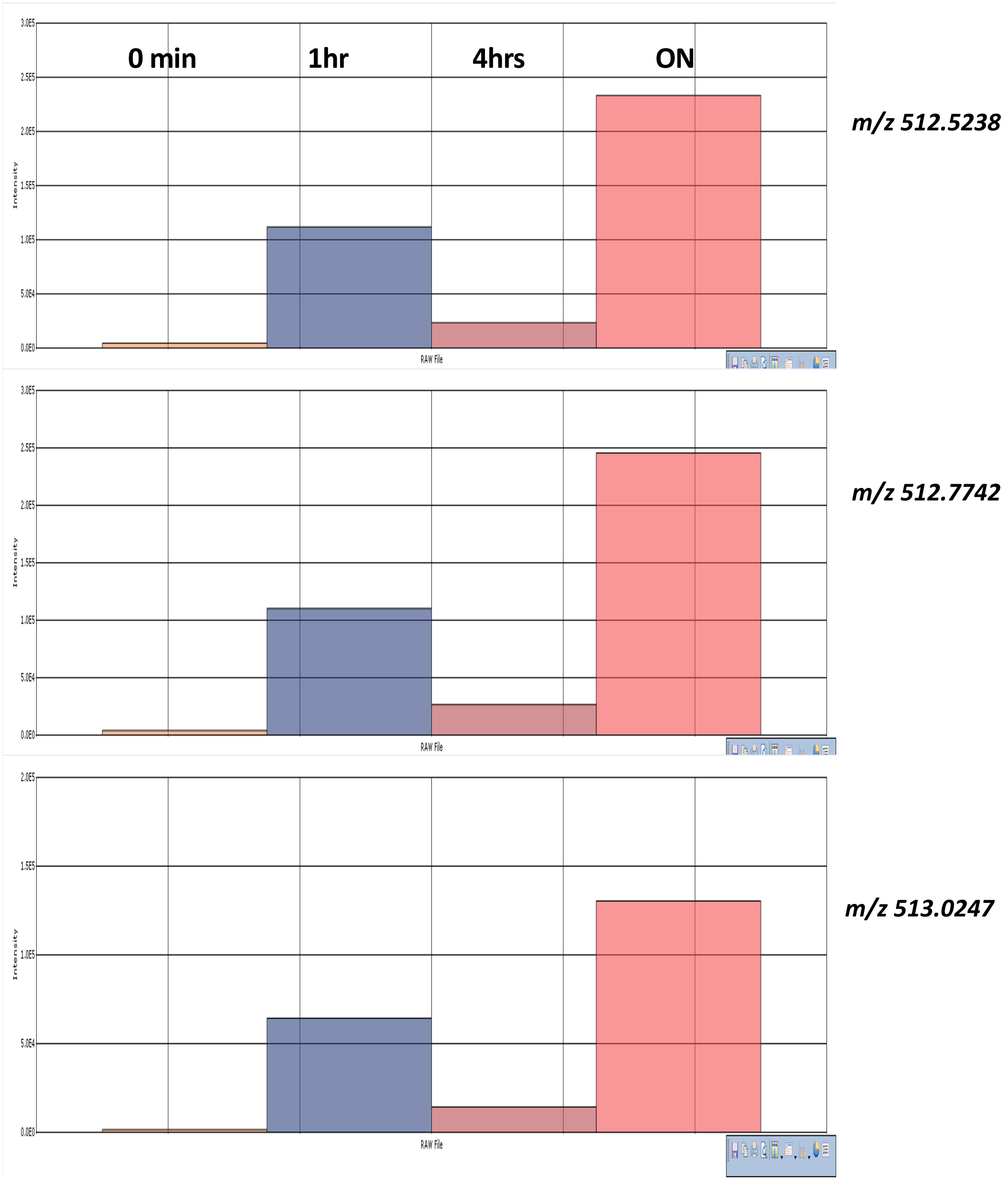

(ADPr)KKPIKGKQAPRKK-NH2
[M+4H]4+

Supplementary Figure 6: Characterization of ADP-ribosylation of SET8 synthetic peptide KKPIKGKQAPRKK-NH2 using high resolution mass spectrometry and isotope quantification in nanoLC. (a) Quantification of peptide KKPIKGKQAPRKK-NH2 (charge +3) at four time points: 0 min (negative control), 1hr, 4 hrs and overnight (ON) reaction with PARP1. Peak areas were determined with SIEVE software package for the three main isotopes m/z 502.67534, m/z 503.00960 and m/z 503.34383, theoretical values determined with Protein Prospector MS Isotope. The figure displays the measured isotopes 0, 1 and 2 with Orbitrap MS. (b) Quantification of peptide (ADPr)KKPIKGKQAPRKK-NH2 (charge +4) at four time points: 0 min (negative control), 1hr, 4 hrs and overnight (ON) reaction with PARP1. Peak areas were determined with SIEVE software package for the three main isotopes m/z 512.52360, m/z 512.77430 and m/z 513.02497, theoretical values determined with Protein Prospector MS Isotope. The figure displays the measured isotopes 0, 1 and 2 with Orbitrap MS.

Fig. S7

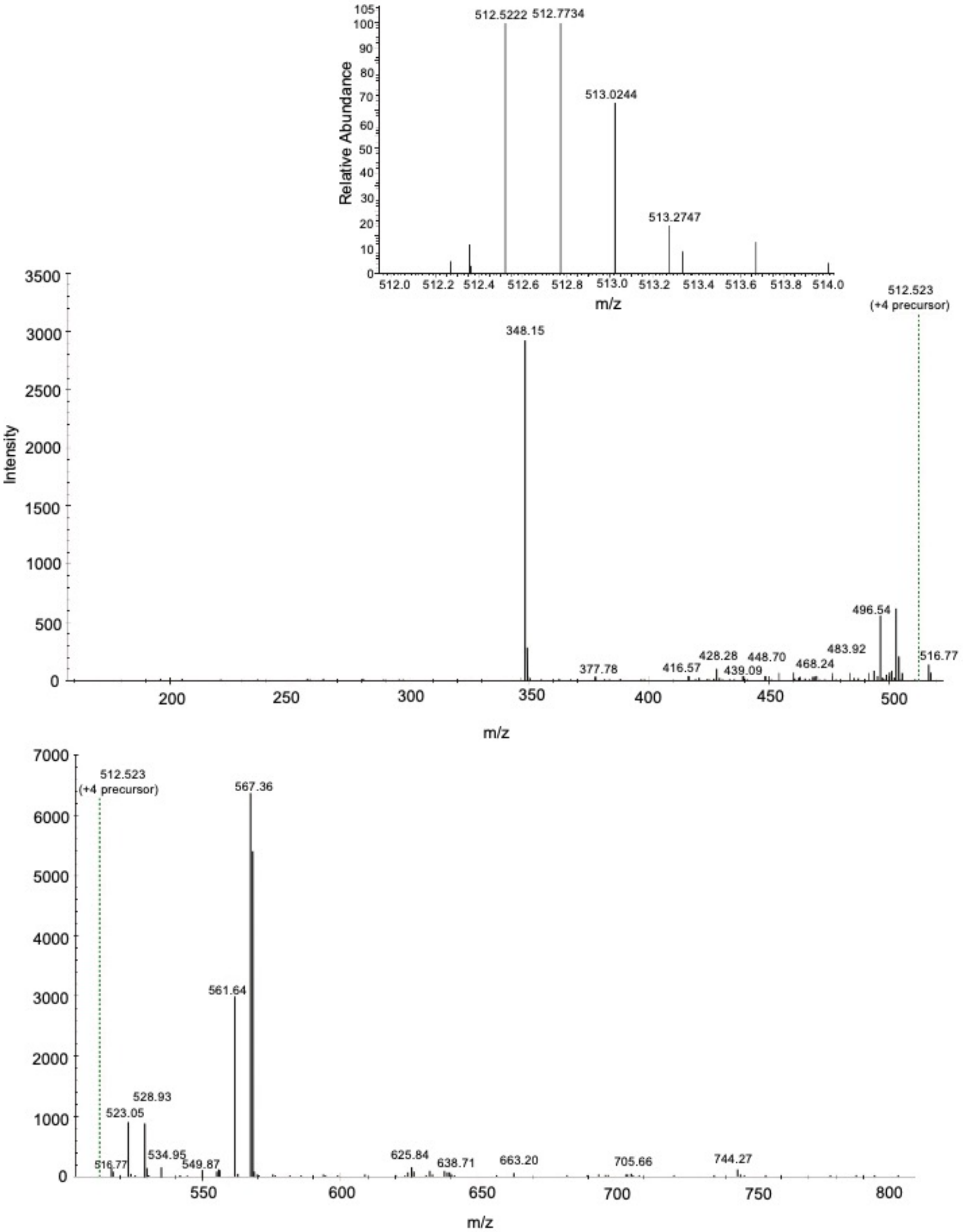

Supplementary Figure 7: Characterization of ADP-ribosylation of SET8 synthetic peptide KKPIKGKQAPRKK-NH2 using MS/MS CID fragmentation, [M+4H]4+ precursor m/z 512.52. Characteristics ADP-ribose modification fragmentation ions (m-ions peptide free modification fragment ions): m5=348 and m8=428 (top panel). Fragment peptide ions are present most probably due to neutral loss of ADP-ribose fragments, within the unit resolution on linear ion trap MS/MS spectra (bottom panel). Inset shows the [M+4H]4+ precursor full scan MS spectrum.

Fig. S8

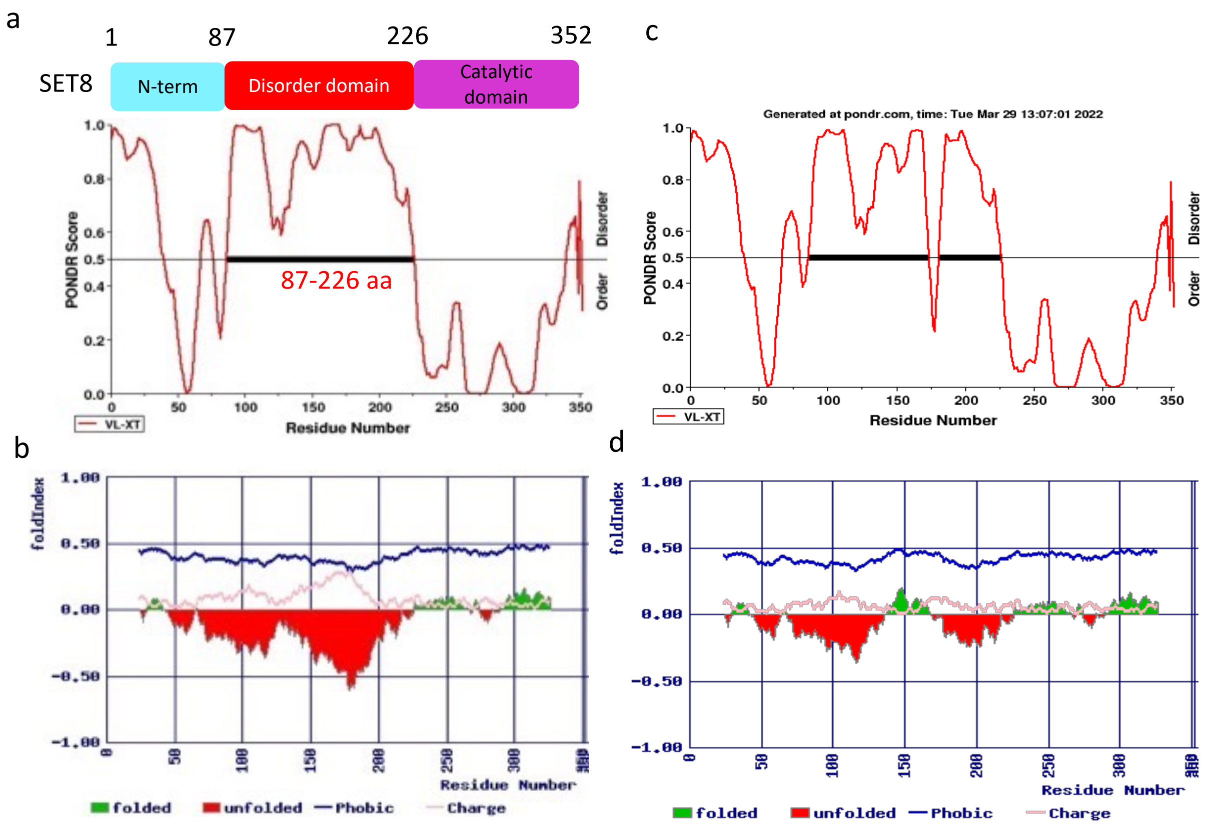

Supplementary Figure 8: Folding Profile of Wild Type SET8 and SET8 mutation. (a) & (c) Structural disordered prediction for SET8 (87-226 aa disordered region) & SET8 mutation using PONDR-VLXT. (b) & (d) Protein folding profiling using FoldIndex tool for SET8 & SET8 mutation (greens are folded and reds are unfolded).

Fig. S9

a **SET8**

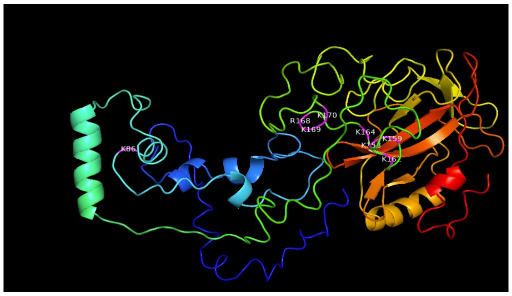

Residue Position: K86, K158, K159, K160, R168, K169, K170, K174

b **SET8 M**

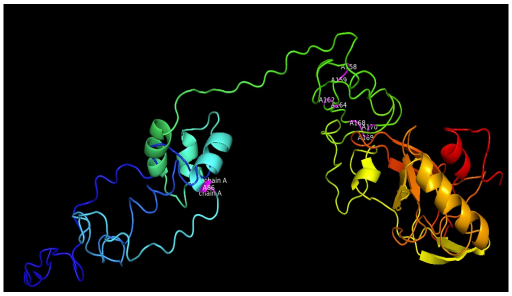

Residue Position: K86A, K158A, K159A, K160A, R168A, K169A, K170A, K174A

c

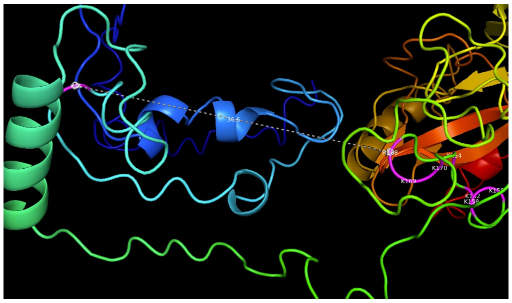

Distance between K86-R168: 36.6A˚

d

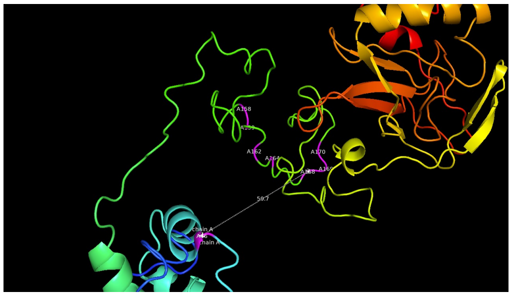

Distance between K86A-R168A: 50.7A˚

e

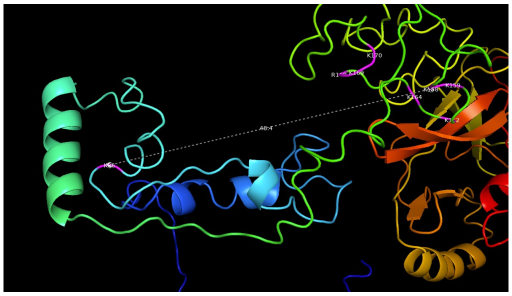

Distance between K86-K158: 48.4A˚

f

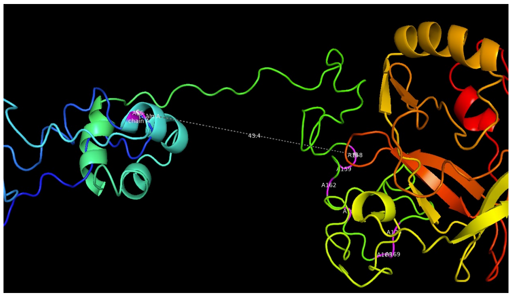

Distance between K86A-K158A: 43.4A˚

Supplementary Figure 9: PDB structures of SET8 and mutated SET8 generated from I-TASSER. (a) PDB structure of Wild Type SET8 protein where lysine enriched domains are marked in pink (K86, K158, K159, K160, K162, R168, K169, K170, K174). (b) PDB structure of Mutated SET8 protein where mutated alanine enriched domains are colored in pink. (c) Distance from K86-R168 = 36.6Å of wild type SET8. (d) Distance from K86A-R168A = 50.7Å of mutated SET8. (e) Distance from K86-K158 = 48.4Å of wild type SET8. (f) Distance from K86A-K158A = 43.4Å of mutated SET8.

Fig. S10

a

SET8

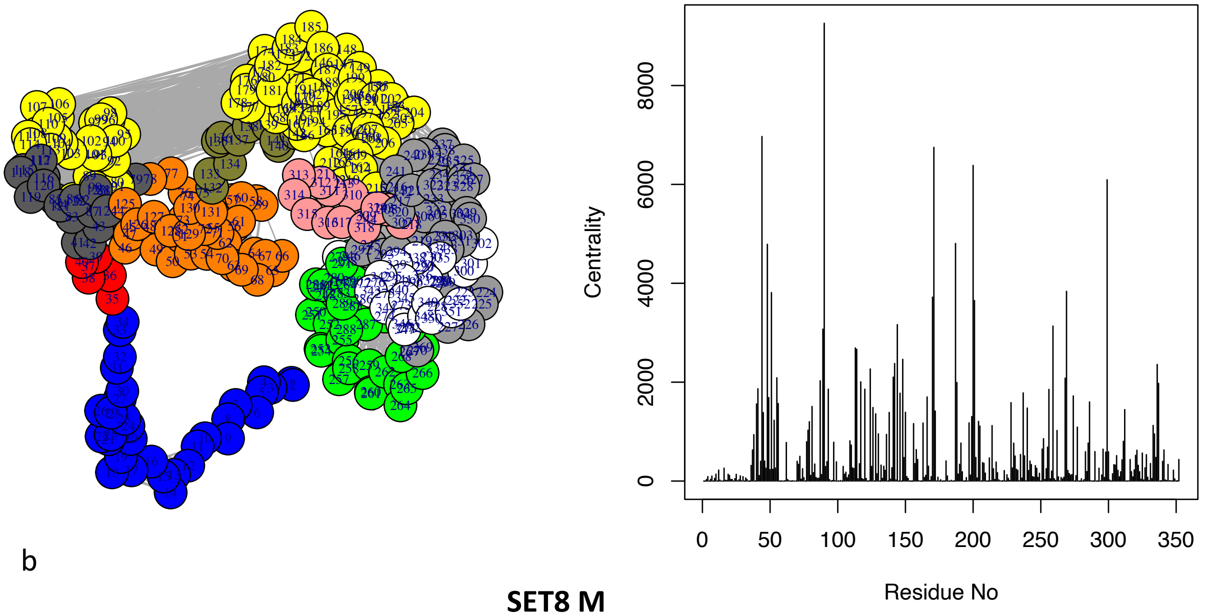

b

SET8 M

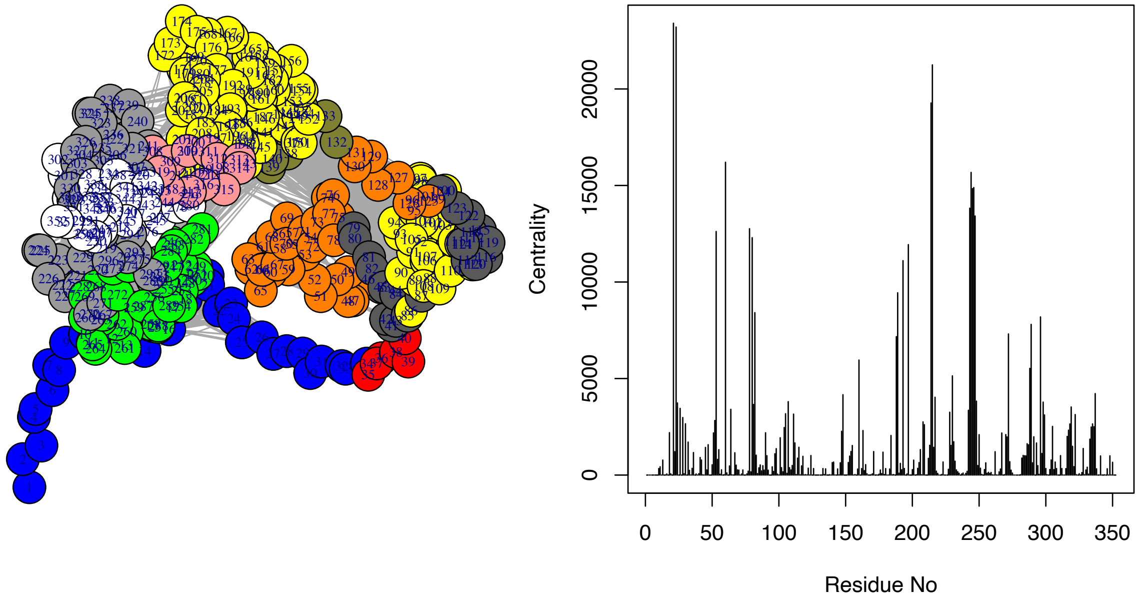

Supplementary Figure 10: Structure Network of the Monomeric Structure of the wildtype and mutated SET8. (a) Structure Network Model (top, right) and Betweenness Centrality Plotting (top, left) throughout residue space for wildtype SET8. (b) Structure Network Model (bottom, right) and Betweenness Centrality Plotting (bottom, left) throughout residue space for mutated SET8.

Fig. S11

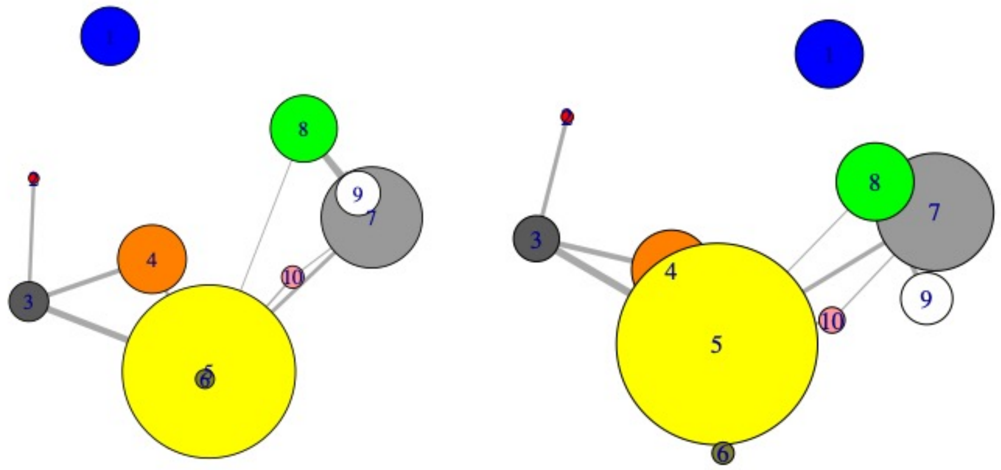

No. of Clusters	No. of Members within Clusters	Residues
1	34	1:34
2	6	35:40
3	23	41:45, 79:85, 87, 115:124
4	40	46:78, 125:131
5	101	86, 88:114, 143:215
6	11	132:142
7	59	216:245, 270:271, 292:299, 303:306, 320:334
8	39	246:269, 272:275, 281:291
9	26	276:280, 300:302, 335:352
10	13	307:319

**No. of Clusters**	**No. of Members within Clusters**	**Residues**
**1**	7	1:7
**2**	1	8
**3**	63	9:22, 246:293, 295
**4**	42	23:30, 46:79
**5**	63	31:45, 80:127
**6**	1	128
**7**	3	129:131
**8**	29	132:160
**9**	54	161:214
**10**	62	15:245, 294, 296:309, 319:334
**11**	9	310:318
**12**	18	335:352

Supplementary Figure 11. Cluster Network Models based on Betweenness Centrality Scoring where wild type SET8 has 10 modules (top, right), mutated SET8 has 12 modules (top, left). The residual members of each cluster are given in Tabular form for wild type SET8 (bottom, right) and mutated SET8 (bottom, left).

Fig. S12

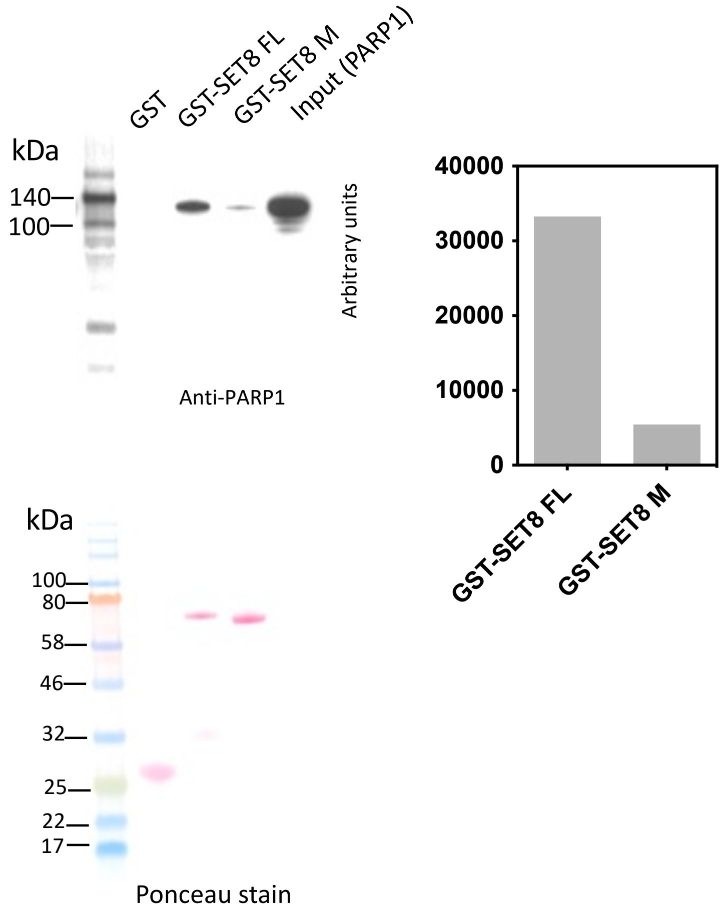

Supplementary Figure 12. SET8 Lysine mutation restrict PARP1 binding. GST pull down of GST-SET8FL or GST-SET8 M (K86A, K158/159A, K162/164A, R168A, K169/170A, K174A) of PARP1 full-length enzyme. Western blot probed with anti-PARP1 antibody (top, left), and the relative pulldown of PARP1 quantitated and shown in arbitrary unit (top, right) (n=2). Loading of the exact blot shown using ponceau staining (bottom).

Fig. S13

a

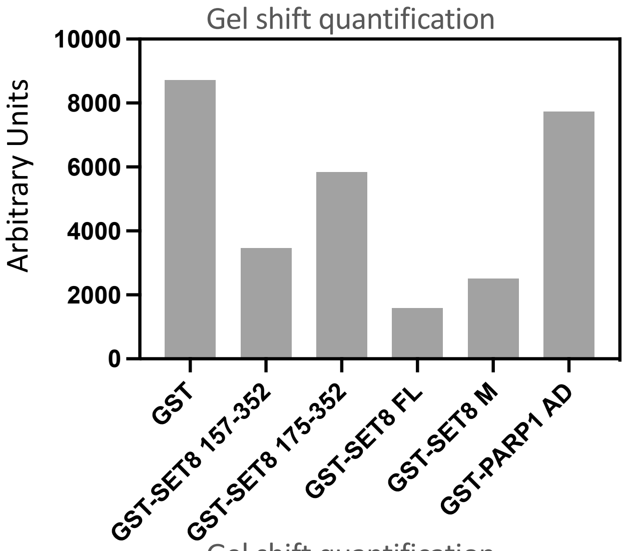

b

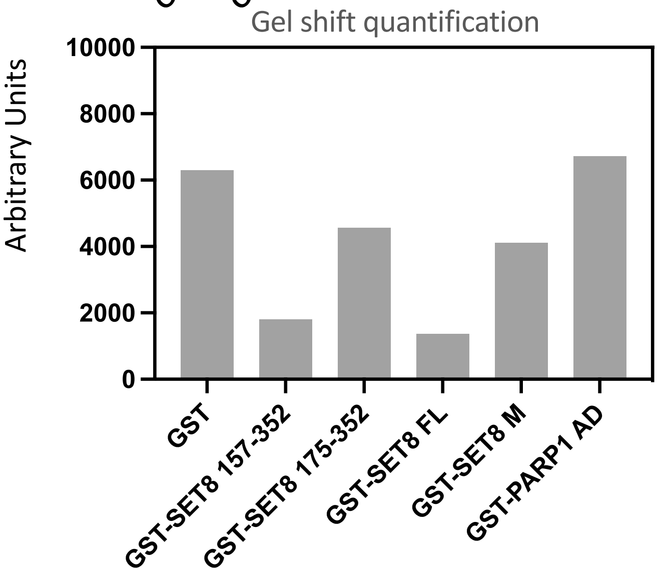

Supplementary Figure 13: Gel shift quantification of shifted DNA with GST-SET8 fusion fragments representing Figure 2a on top (a) (n=2) and Figure 2c at the bottom (b) (n=2).

Fig. S14

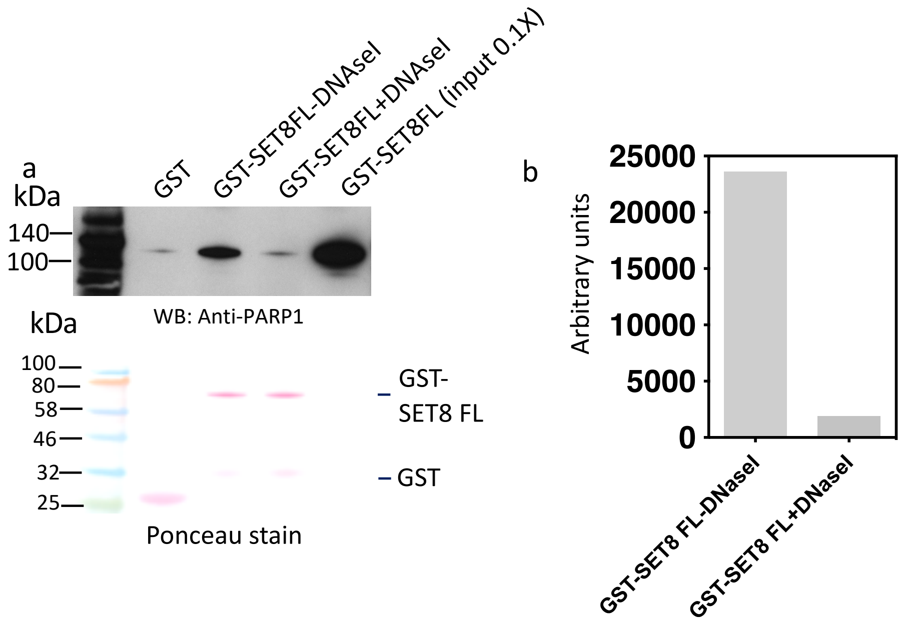

Supplementary Figure 14: DNA enhances SET8 and PARP1 binding. (a) GST pull down assay of GST-SET8 FL with PARP1 full length in the presence or absence of DNase I. Western blot using anti-PARP1 antibody revels the presence of PARP1 (top, left side). (b) The relative pulldown of PARP1 quantitated and shown in arbitrary unit (right side) (n=2). Loading of the exact blot shown using ponceau staining (bottom, left side).

Fig. S15

a

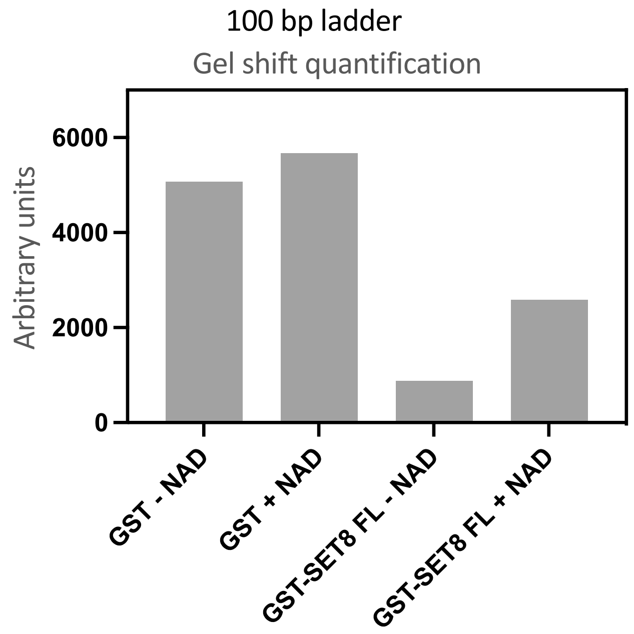

b

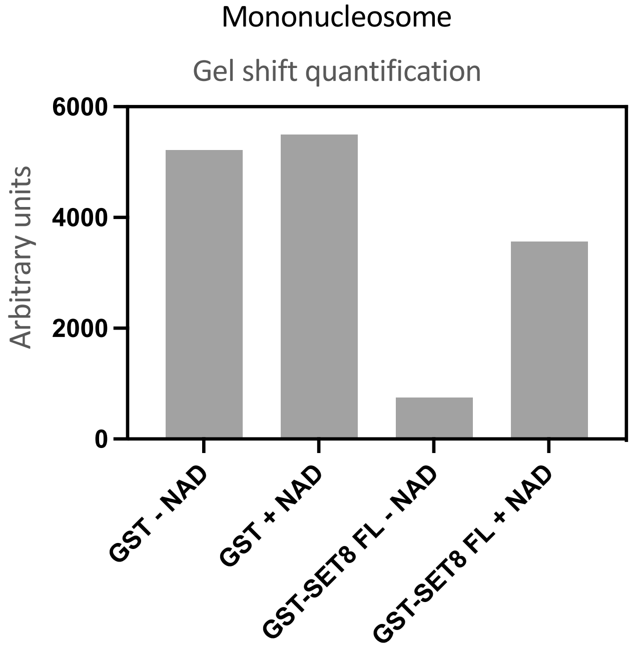

Supplementary Figure 15: Gel shift quantification of shifted DNA with GST-SET8 fusion fragments representing Figure 2d left lanes 2-5 with 100 bp DNA ladder on top (a) (n=2) and right lanes 7-10 at the bottom (b) (n=2).

Fig. S16

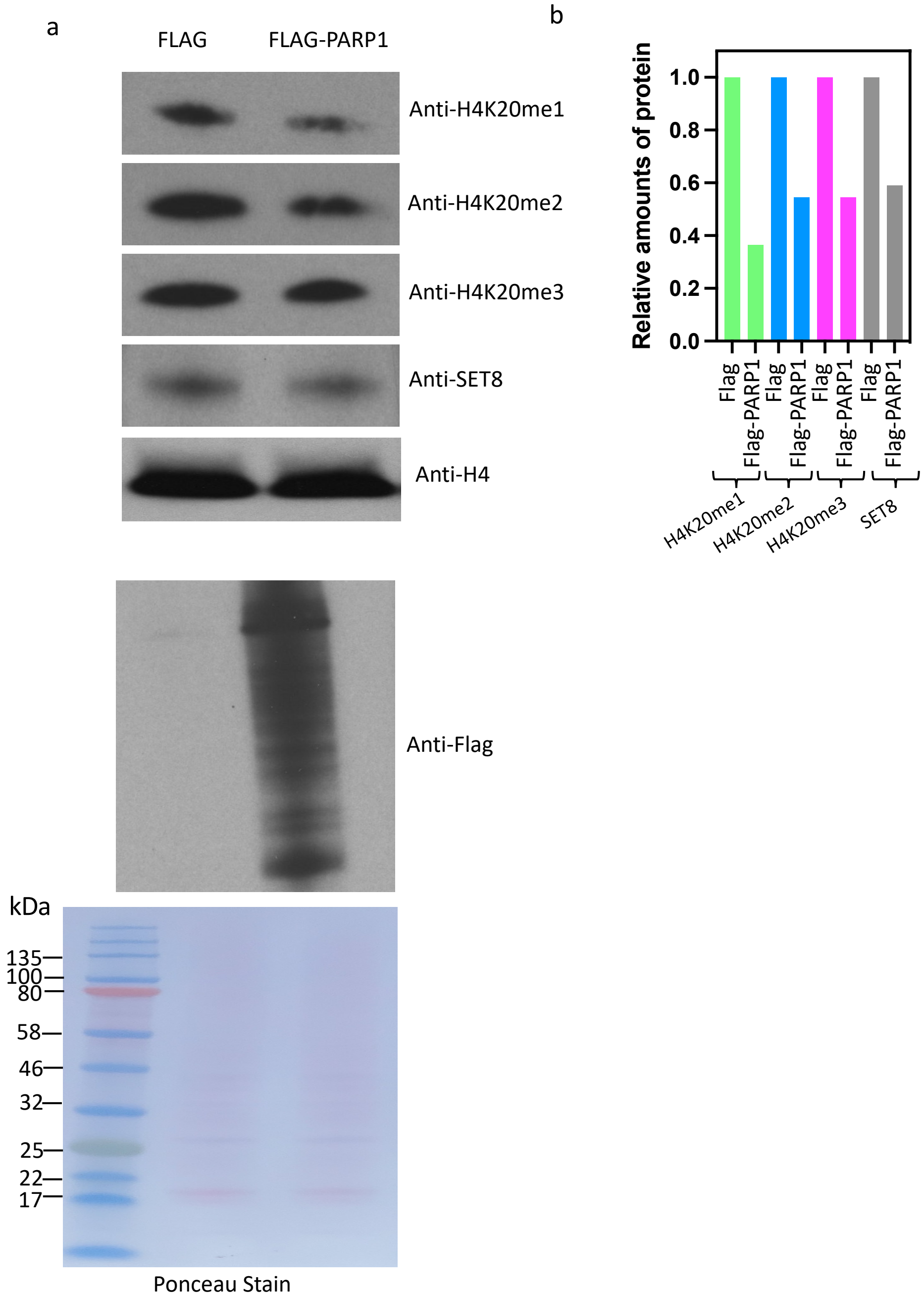

Supplementary Figure 16: PARP1 overexpression leads to change the cellular levels of H4K20 methylation. (a) Equal amounts of cell extract from cells transfected with FLAG or FLAG-PARP1, blotted and probed with antibodies as indicated. Anti-Flag antibody demonstrate auto-parylation of FLAG-PARP1. Equal loading is shown by ponceau staining. (b) Western blot showing levels of mono, di, tri methylation of H4K20, histone H4 and SET8 (n=2). Relative amounts of protein are measured by scanning three independent blots

Fig. S17

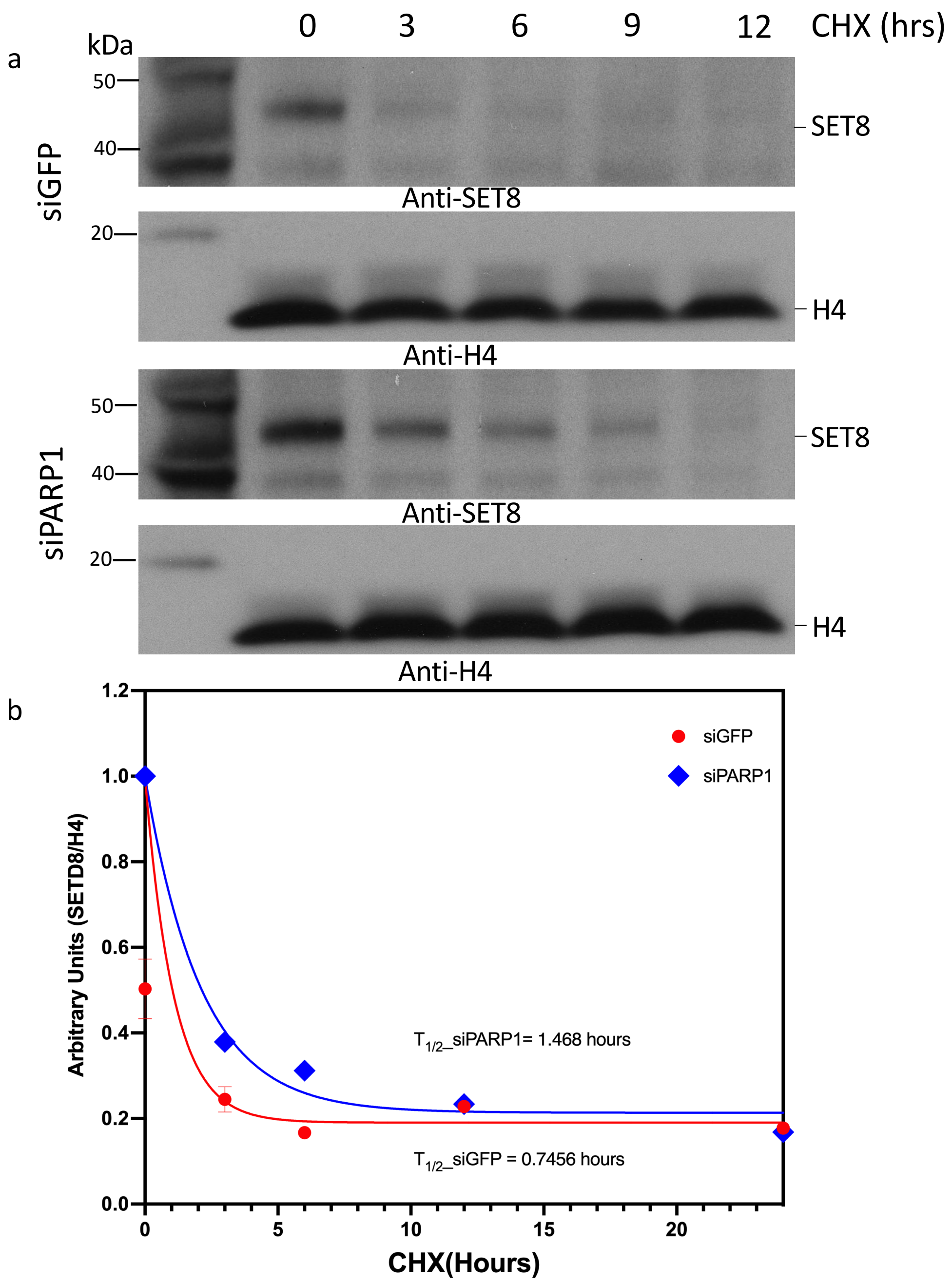

Supplementary Figure 17: Half-life of endogenous SET8 in PARP1 depleted cells. (a) siGFP (control) or siPARP1 was used for knockdown in Hela cells. Hrs of cycloheximide (CHX) is indicated. SET8 and H4 are revealed by western blot using specific antibodies. (b) In the lower panel amount of SET8 per lane is normalized with histone H4 and plotted. Data from two biological experiments are used.

Fig. S18

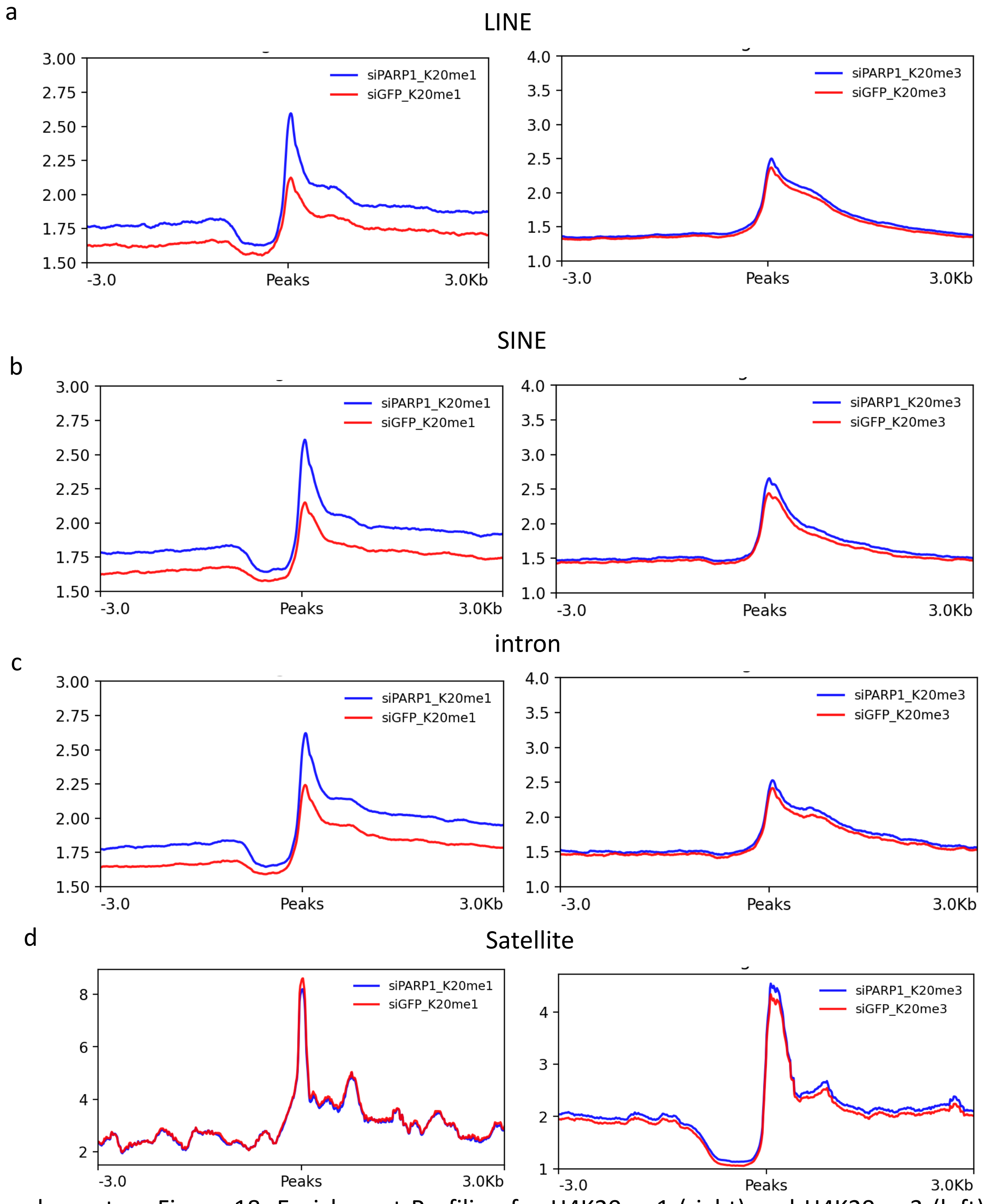

Supplementary Figure 18: Enrichment Profiling for H4K20me1 (right) and H4K20me3 (left) for PARP1 Knockdown and Control Conditions for different Peak Annotating genomic Regions. (a) LINE, (b) SINE, (c) Intron, and (d) Satellite.

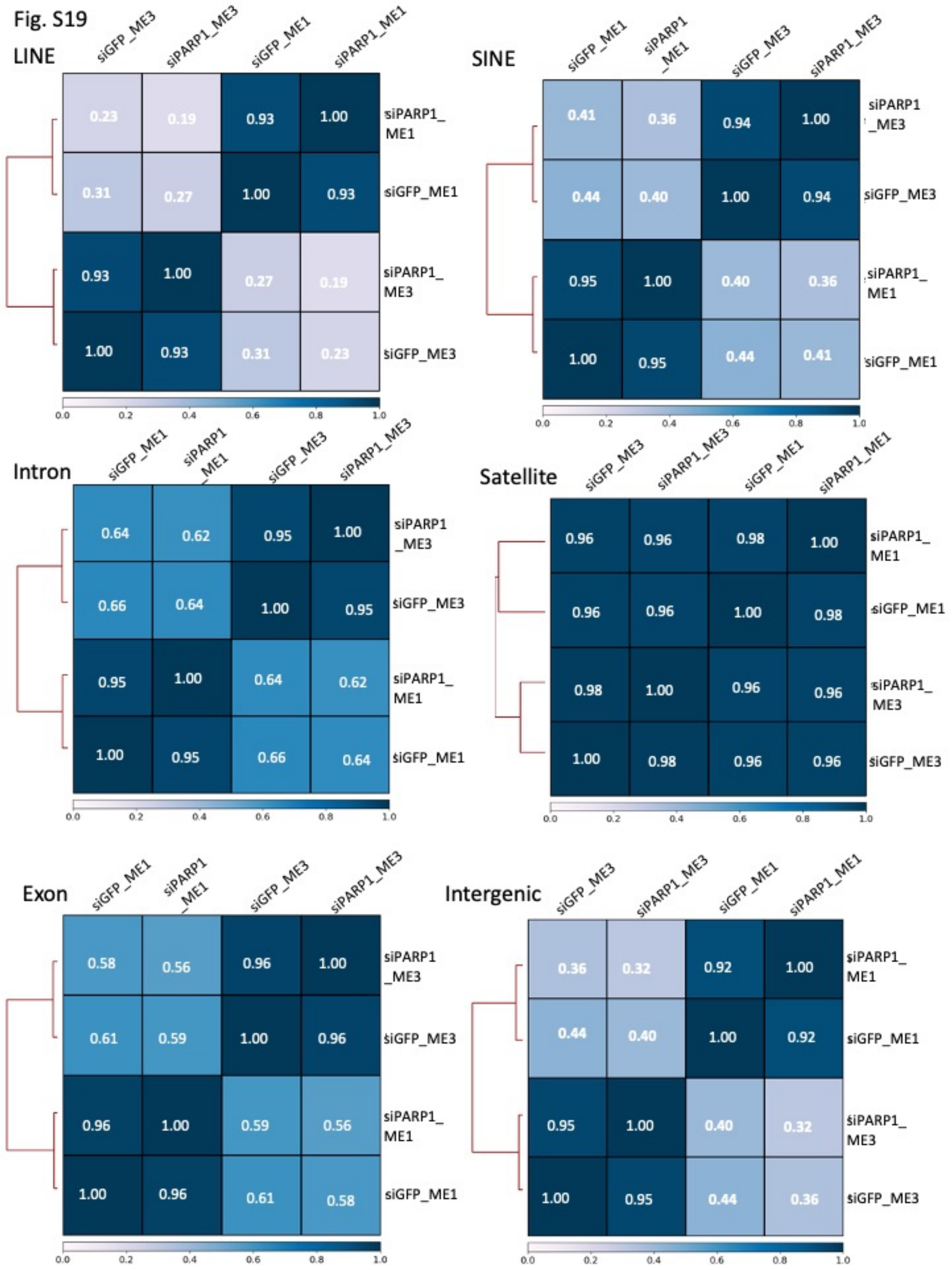

Supplementary Figure 19: Spearman Correlation Heatmaps of PARP1 Knockdown and Control Conditions for different Peak Annotating Regions of H4K20me1 and H4K20me3 i.e., LINE, SINE, Intron, Satellite, Exon, and Intergenic.

Fig. S20

a

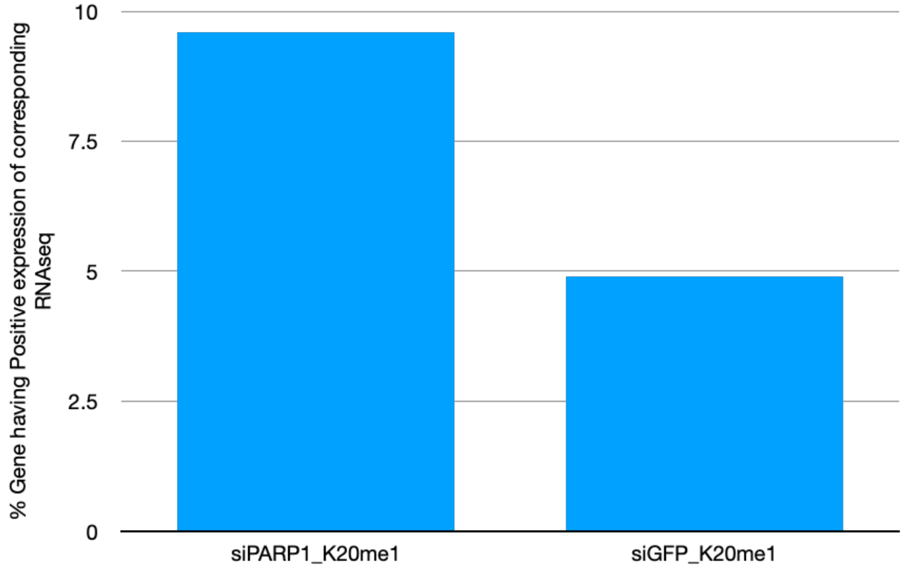

b

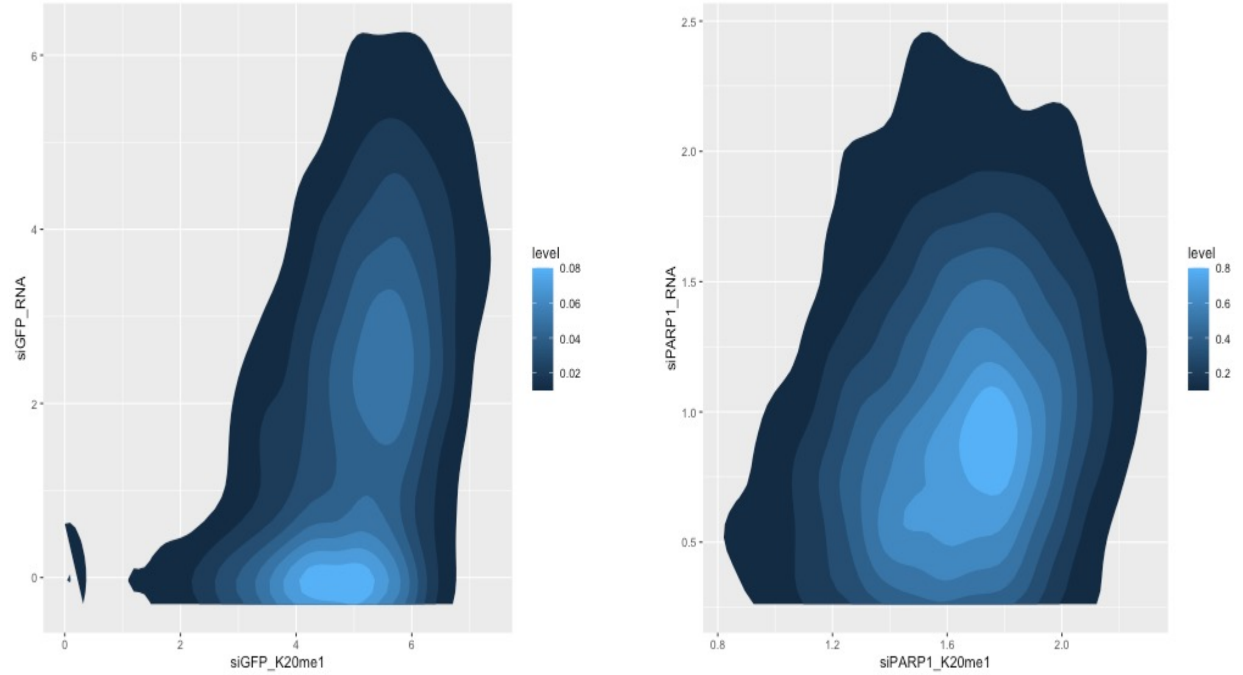

Supplementary Figure 20: Comparative study between RNA seq and CHIP seq from PARP1 Knockdown and Control Conditions. (a) Overlapping genes between intragenic regions from different conditions and corresponding Positive Expression from RNA seq. (b) Density plot Profiling to show the distribution of the expression between siGFP_H4K20me1 positive gene expression and corresponding RNA seq genomic expression profiling (top, left) and, between siPARP1_H4K20me1 positive gene expression and corresponding RNA seq genomic expression profiling (top, right).

Fig. S21

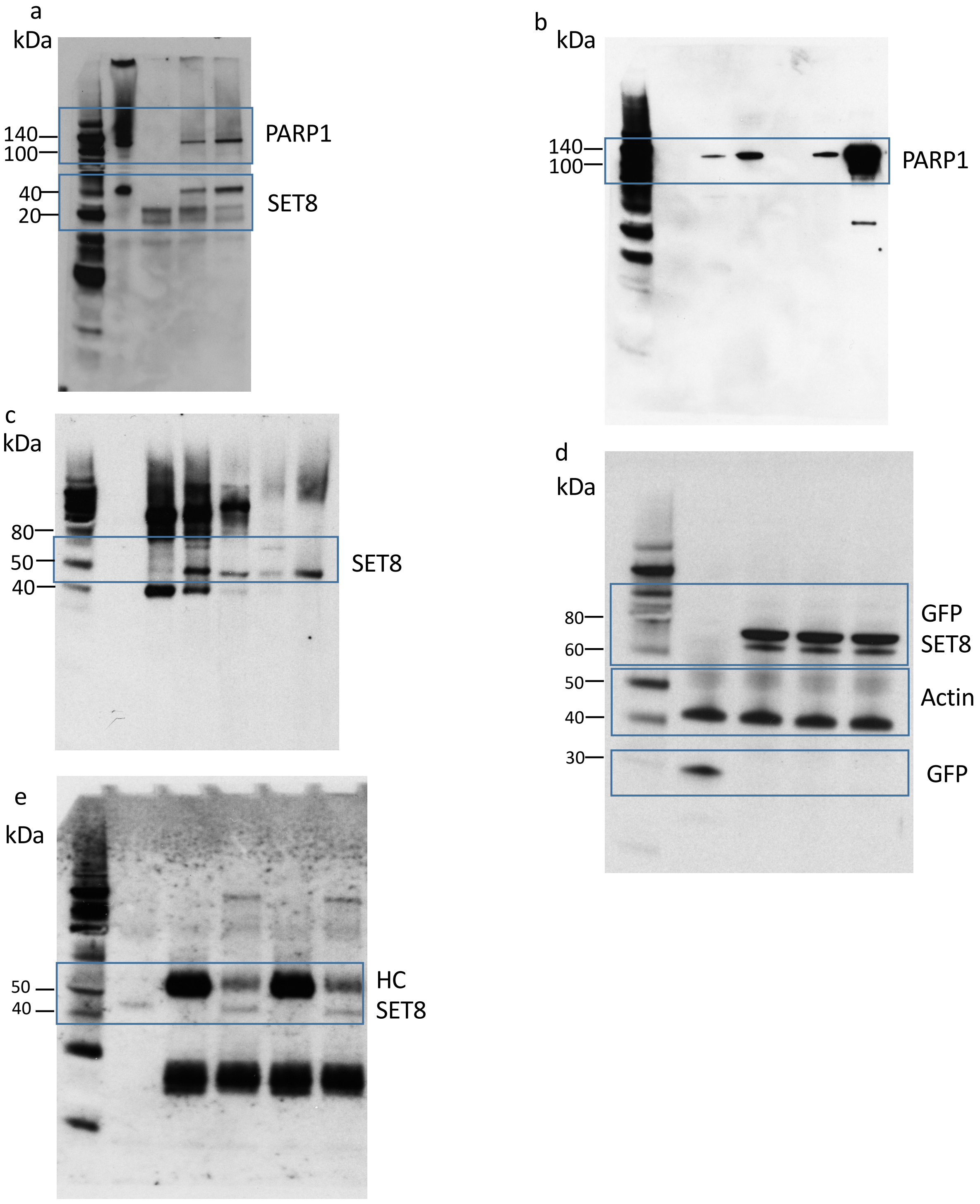

a
kDa
140
100
40
20
PARP1
SET8
b
kDa
140
100
PARP1
c
kDa
80
50
40
SET8
d
kDa
80
60
50
40
30
GFP
SET8
Actin
GFP
e
kDa
50
40
HC
SET8

Fig. S21

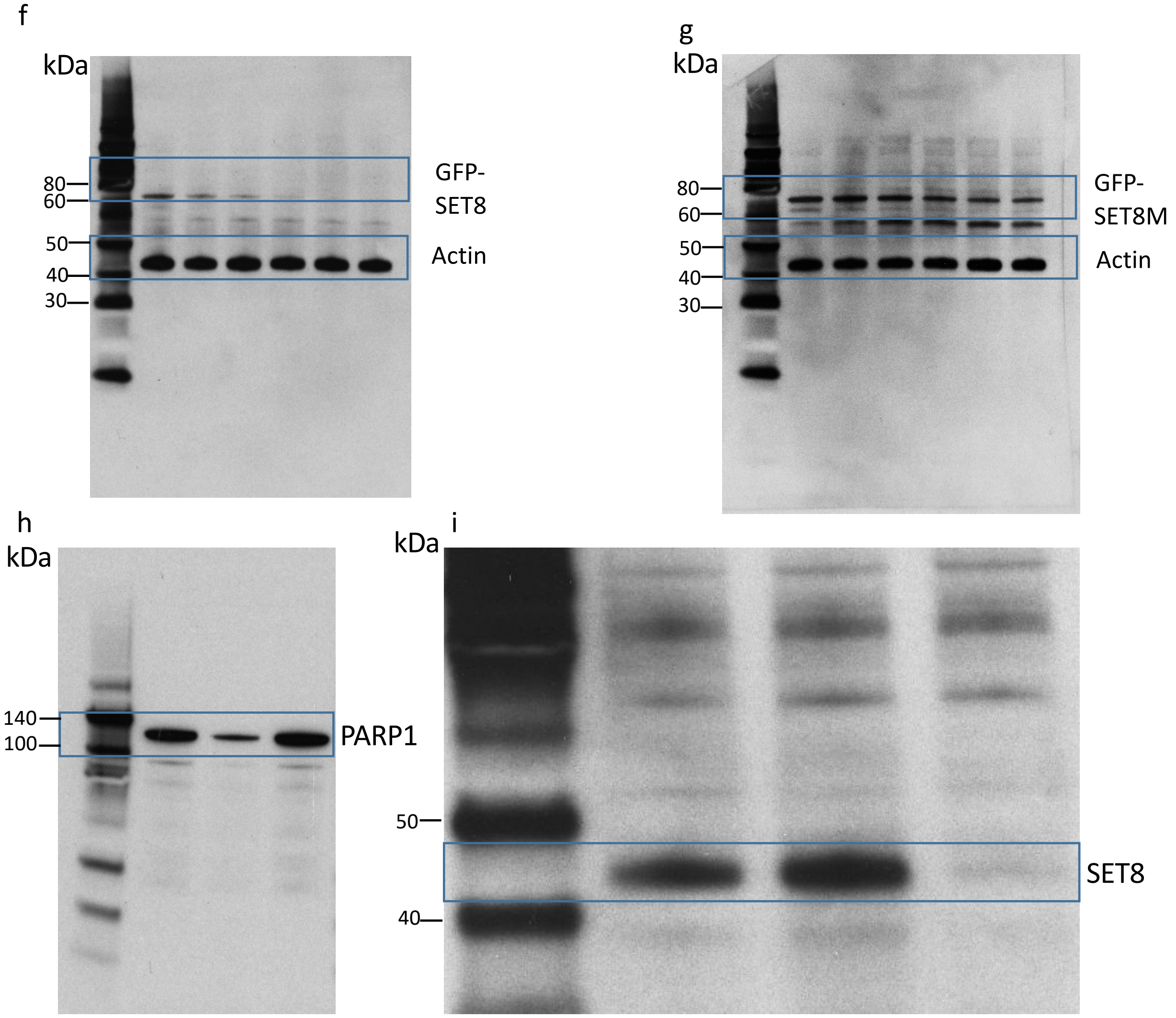

Supplementary Figure 21: Uncropped western blots correspond to Figure 1a,1c-d (a-c) and Figure 3b-c (d-e); Figure 4b (f-g) and Figure 4c (h-i)

Supplementary Table 1: Proteomic analysis of SET8 pull-down in HEK293T cells using LC-MS.							
	Accession	-10lgP	Coverage (%)	#Peptides	#Unique	Avg. Mass	Description
	P09874|PARP1_HUMAN	191.03	30	36	36	113084	Poly [ADP-ribose] polymerase 1 OS=Homo sapiens GN=PARP1 PE=1 SV=4
	Q9NQR1-2|SETD8_HUMAN	179.9	53	29	29	39223	Isoform 2 of N-lysine methyltransferase SETD8 OS=Homo sapiens GN=SETD8
	P07437|TBB5_HUMAN	170.53	51	36	35	49671	Tubulin beta chain OS=Homo sapiens GN=TUBB PE=1 SV=2
	P68363|TBA1B_HUMAN	158.69	49	37	36	50152	Tubulin alpha-1B chain OS=Homo sapiens GN=TUBA1B PE=1 SV=1
	Q00839-2|HNRPU_HUMAN	156.78	26	27	27	88980	Isoform Short of Heterogeneous nuclear ribonucleoprotein U OS=Homo sapiens GN=HNRNPU
	P11498|PYC_HUMAN	150.73	21	30	29	129634	Pyruvate carboxylase mitochondrial OS=Homo sapiens GN=PC PE=1 SV=2
	P08107|HSP71_HUMAN	149.74	31	26	11	70052	Heat shock 70 kDa protein 1A/1B OS=Homo sapiens GN=HSPA1A PE=1 SV=5
	P08238|HS90B_HUMAN	147.66	18	13	8	83264	Heat shock protein HSP 90-beta OS=Homo sapiens GN=HSP90AB1 PE=1 SV=4
	P13639|EF2_HUMAN	147.49	20	22	22	95338	Elongation factor 2 OS=Homo sapiens GN=EEF2 PE=1 SV=4
	P11142|HSP7C_HUMAN	141.56	27	25	11	70898	Heat shock cognate 71 kDa protein OS=Homo sapiens GN=HSPA8 PE=1 SV=1
	Q13263|TIF1B_HUMAN	136.65	17	15	15	88550	Transcription intermediary factor 1-beta OS=Homo sapiens GN=TRIM28 PE=1 SV=5
	P61978-2|HNRPK_HUMAN	131.92	35	20	20	51028	Isoform 2 of Heterogeneous nuclear ribonucleoprotein K OS=Homo sapiens GN=HNRNPK
	P31943|HNRH1_HUMAN	130.44	25	16	7	49229	Heterogeneous nuclear ribonucleoprotein H OS=Homo sapiens GN=HNRNPH1 PE=1 SV=4
	P14866|HNRPL_HUMAN	125.33	30	14	14	64133	Heterogeneous nuclear ribonucleoprotein L OS=Homo sapiens GN=HNRNPL PE=1 SV=2
	P19338|NUCL_HUMAN	123.56	21	16	16	76615	Nucleolin OS=Homo sapiens GN=NCL PE=1 SV=3
	Q96RQ3|MCCA_HUMAN	122.47	14	9	9	80473	Methylcrotonoyl-CoA carboxylase subunit alpha mitochondrial OS=Homo sapiens GN=MCCC1 PE=1 SV=3
	Q92841|DDX17_HUMAN	121.95	17	13	12	80273	Probable ATP-dependent RNA helicase DDX17 OS=Homo sapiens GN=DDX17 PE=1 SV=2
	Q09028-4|RBBP4_HUMAN	121.94	32	10	7	43482	Isoform 4 of Histone-binding protein RBBP4 OS=Homo sapiens GN=RBBP4
	P07900|HS90A_HUMAN	115.99	7	7	3	84660	Heat shock protein HSP 90-alpha OS=Homo sapiens GN=HSP90AA1 PE=1 SV=5
	P05166|PCCB_HUMAN	115.83	17	11	11	58216	Propionyl-CoA carboxylase beta chain mitochondrial OS=Homo sapiens GN=PCCB PE=1 SV=3
	P60709|ACTB_HUMAN	115.26	17	9	9	41737	Actin cytoplasmic 1 OS=Homo sapiens GN=ACTB PE=1 SV=1
	P55795|HNRH2_HUMAN	112.89	14	9	1	49264	Heterogeneous nuclear ribonucleoprotein H2 OS=Homo sapiens GN=HNRNPH2 PE=1 SV=1
	Q12906-5|ILF3_HUMAN	109.29	7	6	6	74607	Isoform 5 of Interleukin enhancer-binding factor 3 OS=Homo sapiens GN=ILF3
	P68104-2|EF1A1_HUMAN	107.81	24	13	13	47869	Isoform 2 of Elongation factor 1-alpha 1 OS=Homo sapiens GN=EEF1A1
	P52272|HNRPM_HUMAN	106.13	14	11	11	77516	Heterogeneous nuclear ribonucleoprotein M OS=Homo sapiens GN=HNRNPM PE=1 SV=3
	P10809|CH60_HUMAN	105.64	11	6	6	61055	60 kDa heat shock protein mitochondrial OS=Homo sapiens GN=HSPD1 PE=1 SV=2
	Q9HCC0|MCCB_HUMAN	101.46	12	9	9	61333	Methylcrotonoyl-CoA carboxylase beta chain mitochondrial OS=Homo sapiens GN=MCCC2 PE=1 SV=1
	P11387|TOP1_HUMAN	100.61	11	6	6	90726	DNA topoisomerase 1 OS=Homo sapiens GN=TOP1 PE=1 SV=2
	Q13838|DX39B_HUMAN	98.93	14	8	8	48991	Spliceosome RNA helicase DDX39B OS=Homo sapiens GN=DDX39B PE=1 SV=1
	P08670|VIME_HUMAN	98.32	18	10	9	53652	Vimentin OS=Homo sapiens GN=VIM PE=1 SV=4
	P23246-2|SFPQ_HUMAN	97.17	14	7	7	72263	Isoform Short of Splicing factor proline- and glutamine-rich OS=Homo sapiens GN=SFPQ
	P12956|XRCC6_HUMAN	94.61	12	7	7	69843	X-ray repair cross-complementing protein 6 OS=Homo sapiens GN=XRCC6 PE=1 SV=2
	P06733|ENOA_HUMAN	93.15	19	9	8	47169	Alpha-enolase OS=Homo sapiens GN=ENO1 PE=1 SV=2
	P05165|PCCA_HUMAN	92.96	12	8	7	80059	Propionyl-CoA carboxylase alpha chain mitochondrial OS=Homo sapiens GN=PCCA PE=1 SV=4
	P26368-2|U2AF2_HUMAN	92.22	17	6	6	53121	Isoform 2 of Splicing factor U2AF 65 kDa subunit OS=Homo sapiens GN=U2AF2

	Q9UMS4|PRP19_HUMAN	91.03	15	7	7	55181	Pre-mRNA-processing factor 19 OS=Homo sapiens GN=PRPF19 PE=1 SV=1
	P26599|PTBP1_HUMAN	89.88	15	7	7	57221	Polypyrimidine tract-binding protein 1 OS=Homo sapiens GN=PTBP1 PE=1 SV=1
	Q8NC51-4|PAIRB_HUMAN	89.65	21	6	6	42427	Isoform 4 of Plasminogen activator inhibitor 1 RNA-binding protein OS=Homo sapiens GN=SERBP1
	P55209-2|NP1L1_HUMAN	88.54	11	5	4	42762	Isoform 2 of Nucleosome assembly protein 1-like 1 OS=Homo sapiens GN=NAP1L1
	P36578|RL4_HUMAN	88.28	23	8	8	47697	60S ribosomal protein L4 OS=Homo sapiens GN=RPL4 PE=1 SV=5
	P35637-2|FUS_HUMAN	86.59	9	5	5	53355	Isoform Short of RNA-binding protein FUS OS=Homo sapiens GN=FUS
	P13010|XRCC5_HUMAN	86.05	8	6	6	82705	X-ray repair cross-complementing protein 5 OS=Homo sapiens GN=XRCC5 PE=1 SV=3
	O75475|PSIP1_HUMAN	83.81	12	5	5	60103	PC4 and SFRS1-interacting protein OS=Homo sapiens GN=PSIP1 PE=1 SV=1
	O43390-3|HNRPR_HUMAN	83.26	13	7	7	66980	Isoform 3 of Heterogeneous nuclear ribonucleoprotein R OS=Homo sapiens GN=HNRNPR
	Q16576-2|RBBP7_HUMAN	82.04	12	4	2	52314	Isoform 2 of Histone-binding protein RBBP7 OS=Homo sapiens GN=RBBP7
	P67809|YBOX1_HUMAN	78.03	13	3	3	35924	Nuclease-sensitive element-binding protein 1 OS=Homo sapiens GN=YBX1 PE=1 SV=3
	P39023|RL3_HUMAN	77.63	14	5	5	46109	60S ribosomal protein L3 OS=Homo sapiens GN=RPL3 PE=1 SV=2
	P38646|GRP75_HUMAN	77.62	10	7	4	73681	Stress-70 protein mitochondrial OS=Homo sapiens GN=HSPA9 PE=1 SV=2
	P52597|HNRPF_HUMAN	75.88	12	5	2	45672	Heterogeneous nuclear ribonucleoprotein F OS=Homo sapiens GN=HNRNPF PE=1 SV=3
	P14618-2|KPYM_HUMAN	73.4	9	5	5	58062	Isoform M1 of Pyruvate kinase PKM OS=Homo sapiens GN=PKM
	P09651-3|ROA1_HUMAN	70.09	10	4	2	29386	Isoform 2 of Heterogeneous nuclear ribonucleoprotein A1 OS=Homo sapiens GN=HNRNPA1
	Q92945|FUBP2_HUMAN	68.99	6	5	4	73115	Far upstream element-binding protein 2 OS=Homo sapiens GN=KHSRP PE=1 SV=4
	P78371|TCPB_HUMAN	67	6	3	3	57488	T-complex protein 1 subunit beta OS=Homo sapiens GN=CCT2 PE=1 SV=4
	Q9BSJ8|ESYT1_HUMAN	66.27	3	3	3	122856	Extended synaptotagmin-1 OS=Homo sapiens GN=ESYT1 PE=1 SV=1
	Q15233-2|NONO_HUMAN	65.65	7	2	2	43866	Isoform 2 of Non-POU domain-containing octamer-binding protein OS=Homo sapiens GN=NONO
	Q9Y230|RUVB2_HUMAN	65.19	8	4	4	51157	RuvB-like 2 OS=Homo sapiens GN=RUVBL2 PE=1 SV=3
	Q99832-3|TCPH_HUMAN	64.64	5	3	3	54804	Isoform 3 of T-complex protein 1 subunit eta OS=Homo sapiens GN=CCT7
	P55060-4|XPO2_HUMAN	64.23	2	2	2	103879	Isoform 4 of Exportin-2 OS=Homo sapiens GN=CSE1L
	Q9Y265-2|RUVB1_HUMAN	64.06	10	4	4	42127	Isoform 2 of RuvB-like 1 OS=Homo sapiens GN=RUVBL1
	P25205|MCM3_HUMAN	63.14	5	3	3	90981	DNA replication licensing factor MCM3 OS=Homo sapiens GN=MCM3 PE=1 SV=3
	P43243|MATR3_HUMAN	60.57	4	2	2	94623	Matrin-3 OS=Homo sapiens GN=MATR3 PE=1 SV=2
	P11586|C1TC_HUMAN	60.29	4	3	3	101559	C-1-tetrahydrofolate synthase cytoplasmic OS=Homo sapiens GN=MTHFD1 PE=1 SV=3
	P11021|GRP78_HUMAN	59.23	6	5	1	72333	78 kDa glucose-regulated protein OS=Homo sapiens GN=HSPA5 PE=1 SV=2
	P22626-2|ROA2_HUMAN	59.04	7	3	1	36006	Isoform A2 of Heterogeneous nuclear ribonucleoproteins A2/B1 OS=Homo sapiens GN=HNRNPA2B1
	P49368-2|TCPG_HUMAN	58.22	7	3	3	56431	Isoform 2 of T-complex protein 1 subunit gamma OS=Homo sapiens GN=CCT3
	P25705-2|ATPA_HUMAN	58.09	5	2	2	54494	Isoform 2 of ATP synthase subunit alpha mitochondrial OS=Homo sapiens GN=ATP5A1
	Q9BXP5-5|SRRT_HUMAN	57.9	5	3	3	96223	Isoform 5 of Serrate RNA effector molecule homolog OS=Homo sapiens GN=SRRT
	Q9P258|RCC2_HUMAN	57.47	7	3	3	56085	Protein RCC2 OS=Homo sapiens GN=RCC2 PE=1 SV=2
	Q96AE4|FUBP1_HUMAN	55.94	5	3	2	67560	Far upstream element-binding protein 1 OS=Homo sapiens GN=FUBP1 PE=1 SV=3
	P06748-3|NPM_HUMAN	55.73	8	2	2	28400	Isoform 3 of Nucleophosmin OS=Homo sapiens GN=NPM1
	P08621-2|RU17_HUMAN	55.25	4	2	2	50618	Isoform 2 of U1 small nuclear ribonucleoprotein 70 kDa OS=Homo sapiens GN=SNRNP70
	P62987|RL40_HUMAN	54.55	11	2	2	14728	Ubiquitin-60S ribosomal protein L40 OS=Homo sapiens GN=UBA52 PE=1 SV=2
	O60341|KDM1A_HUMAN	54.39	4	2	2	92903	Lysine-specific histone demethylase 1A OS=Homo sapiens GN=KDM1A PE=1 SV=2
	Q99733|NP1L4_HUMAN	52.22	5	2	1	42823	Nucleosome assembly protein 1-like 4 OS=Homo sapiens GN=NAP1L4 PE=1 SV=1

	P20700|LMNB1_HUMAN	51.85	2	2	2	66408	Lamin-B1 OS=Homo sapiens GN=LMNB1 PE=1 SV=2
	Q9Y5B9|SP16H_HUMAN	48.33	2	2	2	119914	FACT complex subunit SPT16 OS=Homo sapiens GN=SUPT16H PE=1 SV=1
	Q14241|ELOA1_HUMAN	47.14	2	2	2	89909	Transcription elongation factor B polypeptide 3 OS=Homo sapiens GN=TCEB3 PE=1 SV=2
	P62805|H4_HUMAN	45.15	30	3	3	11367	Histone H4 OS=Homo sapiens GN=HIST1H4A PE=1 SV=2
	P17987|TCPA_HUMAN	44.76	4	2	2	60344	T-complex protein 1 subunit alpha OS=Homo sapiens GN=TCP1 PE=1 SV=1
	P27694|RFA1_HUMAN	44.53	3	2	2	68138	Replication protein A 70 kDa DNA-binding subunit OS=Homo sapiens GN=RPA1 PE=1 SV=2
	P57053|H2BFS_HUMAN	44.04	8	2	2	13944	Histone H2B type F-S OS=Homo sapiens GN=H2BFS PE=1 SV=2
	P16403|H12_HUMAN	43.14	4	1	1	21365	Histone H1.2 OS=Homo sapiens GN=HIST1H1C PE=1 SV=2
	Q14566|MCM6_HUMAN	42.98	1	1	1	92889	DNA replication licensing factor MCM6 OS=Homo sapiens GN=MCM6 PE=1 SV=1
	O43175|SERA_HUMAN	41.07	4	2	2	56651	D-3-phosphoglycerate dehydrogenase OS=Homo sapiens GN=PHGDH PE=1 SV=4
	P50991|TCPD_HUMAN	40.87	2	1	1	57924	T-complex protein 1 subunit delta OS=Homo sapiens GN=CCT4 PE=1 SV=4
	O60506-4|HNRPQ_HUMAN	40.58	2	1	1	58736	Isoform 4 of Heterogeneous nuclear ribonucleoprotein Q OS=Homo sapiens GN=SYNCRIP
	P13929-2|ENOB_HUMAN	39.88	6	2	1	44115	Isoform 2 of Beta-enolase OS=Homo sapiens GN=ENO3
	P12277|KCRB_HUMAN	39.26	4	2	2	42644	Creatine kinase B-type OS=Homo sapiens GN=CKB PE=1 SV=1
	P42166|LAP2A_HUMAN	38.5	3	2	1	75492	Lamina-associated polypeptide 2 isoform alpha OS=Homo sapiens GN=TMPO PE=1 SV=2
	Q3SY69|AL1L2_HUMAN	38.43	2	1	1	101746	Mitochondrial 10-formyltetrahydrofolate dehydrogenase OS=Homo sapiens GN=ALDH1L2 PE=1 SV=2
	P35659|DEK_HUMAN	38.39	7	2	2	42674	Protein DEK OS=Homo sapiens GN=DEK PE=1 SV=1
	P22314-2|UBA1_HUMAN	37.28	1	1	1	113800	Isoform 2 of Ubiquitin-like modifier-activating enzyme 1 OS=Homo sapiens GN=UBA1
	P55072|TERA_HUMAN	36.9	2	1	1	89322	Transitional endoplasmic reticulum ATPase OS=Homo sapiens GN=VCP PE=1 SV=4
	Q1KMD3|HNRL2_HUMAN	36.76	2	1	1	85105	Heterogeneous nuclear ribonucleoprotein U-like protein 2 OS=Homo sapiens GN=HNRNPUL2 PE=1 SV=1
	P11940-2|PABP1_HUMAN	36.31	2	1	1	61181	Isoform 2 of Polyadenylate-binding protein 1 OS=Homo sapiens GN=PABPC1
	Q53GS9-3|SNUT2_HUMAN	35.42	2	1	1	56359	Isoform 3 of U4/U6.U5 tri-snRNP-associated protein 2 OS=Homo sapiens GN=USP39
	P42167|LAP2B_HUMAN	34.34	4	2	1	50670	Lamina-associated polypeptide 2 isoforms beta/gamma OS=Homo sapiens GN=TMPO PE=1 SV=2
	Q9Y383-3|LC7L2_HUMAN	34.05	3	1	1	46229	Isoform 3 of Putative RNA-binding protein Luc7-like 2 OS=Homo sapiens GN=LUC7L2
	Q14974|IMB1_HUMAN	32.68	1	1	1	97170	Importin subunit beta-1 OS=Homo sapiens GN=KPNB1 PE=1 SV=2
	Q9HAV4|XPO5_HUMAN	32.09	1	1	1	136311	Exportin-5 OS=Homo sapiens GN=XPO5 PE=1 SV=1
	Q13085-3|ACACA_HUMAN	31.92	1	1	1	257236	Isoform 3 of Acetyl-CoA carboxylase 1 OS=Homo sapiens GN=ACACA
	Q5SSJ5|HP1B3_HUMAN	31.7	1	1	1	61207	Heterochromatin protein 1-binding protein 3 OS=Homo sapiens GN=HP1BP3 PE=1 SV=1
	P14868-2|SYDC_HUMAN	31.43	2	1	1	45771	Isoform 2 of Aspartate--tRNA ligase cytoplasmic OS=Homo sapiens GN=DARS
	Q13428-2|TCOF_HUMAN	31.11	1	1	1	144314	Isoform 2 of Treacle protein OS=Homo sapiens GN=TCOF1
	P35499|SCN4A_HUMAN	30.65	1	1	1	208060	Sodium channel protein type 4 subunit alpha OS=Homo sapiens GN=SCN4A PE=1 SV=4
	Q92499-2|DDX1_HUMAN	30.1	1	1	1	69556	Isoform 2 of ATP-dependent RNA helicase DDX1 OS=Homo sapiens GN=DDX1
	Q8WXF1-2|PSPC1_HUMAN	29.28	4	1	1	45571	Isoform 2 of Paraspeckle component 1 OS=Homo sapiens GN=PSPC1
	P11182|ODB2_HUMAN	29.14	2	1	1	53487	Lipoamide acyltransferase component of branched-chain alpha-keto acid dehydrogenase complex mitochondrial OS=Homo sapiens GN=DBT PE=1 SV=3
	P11216|PYGB_HUMAN	28.81	2	1	1	96696	Glycogen phosphorylase brain form OS=Homo sapiens GN=PYGB PE=1 SV=5
	Q86V81|THOC4_HUMAN	28.39	4	1	1	26888	THO complex subunit 4 OS=Homo sapiens GN=ALYREF PE=1 SV=3
	P52209-2|6PGD_HUMAN	28.31	2	1	1	51872	Isoform 2 of 6-phosphogluconate dehydrogenase decarboxylating OS=Homo sapiens GN=PGD
	Q6MZZ7-2|CAN13_HUMAN	28.3	2	1	1	48003	Isoform 2 of Calpain-13 OS=Homo sapiens GN=CAPN13

	Q9Y2L1-2|RRP44_HUMAN	25.82	1	1	1	105040	Isoform 2 of Exosome complex exonuclease RRP44 OS=Homo sapiens GN=DIS3
	Q9NVP1|DDX18_HUMAN	24.44	1	1	1	75407	ATP-dependent RNA helicase DDX18 OS=Homo sapiens GN=DDX18 PE=1 SV=2
	P68431|H31_HUMAN	24.35	6	1	1	15404	Histone H3.1 OS=Homo sapiens GN=HIST1H3A PE=1 SV=2
	P24468-3|COT2_HUMAN	23.86	4	1	1	29163	Isoform 3 of COUP transcription factor 2 OS=Homo sapiens GN=NR2F2
	P20264|PO3F3_HUMAN	23.82	3	1	1	50327	POU domain class 3 transcription factor 3 OS=Homo sapiens GN=POU3F3 PE=2 SV=2
	O00203-3|AP3B1_HUMAN	23.81	1	1	1	116195	Isoform 2 of AP-3 complex subunit beta-1 OS=Homo sapiens GN=AP3B1
	Q9ULU8-2|CAPS1_HUMAN	23.8	1	1	1	144083	Isoform 2 of Calcium-dependent secretion activator 1 OS=Homo sapiens GN=CADPS
	Q93009-3|UBP7_HUMAN	23.67	1	1	1	126269	Isoform 3 of Ubiquitin carboxyl-terminal hydrolase 7 OS=Homo sapiens GN=USP7
	O75444|MAF_HUMAN	22.98	5	1	1	38492	Transcription factor Maf OS=Homo sapiens GN=MAF PE=1 SV=2
	P20023-2|CR2_HUMAN	22.83	1	1	1	110408	Isoform B of Complement receptor type 2 OS=Homo sapiens GN=CR2
	Q96SB4-4|SRPK1_HUMAN	22.5	1	1	1	72384	Isoform 3 of SRSF protein kinase 1 OS=Homo sapiens GN=SRPK1
	Q86VP6|CAND1_HUMAN	22.17	1	1	1	136375	Cullin-associated NEDD8-dissociated protein 1 OS=Homo sapiens GN=CAND1 PE=1 SV=2
	Q15782-5|CH3L2_HUMAN	22	4	1	1	34633	Isoform 2 of Chitinase-3-like protein 2 OS=Homo sapiens GN=CHI3L2
	Q9NZI8|IF2B1_HUMAN	21.92	2	1	1	63481	Insulin-like growth factor 2 mRNA-binding protein 1 OS=Homo sapiens GN=IGF2BP1 PE=1 SV=2
	Q8NFD5|ARI1B_HUMAN	21.87	1	1	1	236121	AT-rich interactive domain-containing protein 1B OS=Homo sapiens GN=ARID1B PE=1 SV=2
	P56192-2|SYMC_HUMAN	21.67	2	1	1	61816	Isoform 2 of Methionine--tRNA ligase cytoplasmic OS=Homo sapiens GN=MARS
	O94842-2|TOX4_HUMAN	21.62	3	1	1	63821	Isoform 2 of TOX high mobility group box family member 4 OS=Homo sapiens GN=TOX4
	P54802|ANAG_HUMAN	21.02	1	1	1	82266	Alpha-N-acetylglucosaminidase OS=Homo sapiens GN=NAGLU PE=1 SV=2
	Q96F45-2|ZN503_HUMAN	20.31	3	1	1	59292	Isoform 2 of Zinc finger protein 503 OS=Homo sapiens GN=ZNF503
	Q9P2K5-2|MYEF2_HUMAN	20.07	2	1	1	61929	Isoform 2 of Myelin expression factor 2 OS=Homo sapiens GN=MYEF2
	Q96KP4|CNDP2_HUMAN	20.04	3	1	1	52879	Cytosolic non-specific dipeptidase OS=Homo sapiens GN=CNDP2 PE=1 SV=2
	Q9Y566-3|SHAN1_HUMAN	20.03	1	1	1	224129	Isoform 3 of SH3 and multiple ankyrin repeat domains protein 1 OS=Homo sapiens GN=SHANK1
	Q7Z5P9-2|MUC19_HUMAN	20.01	0	1	1	564168	Isoform 2 of Mucin-19 OS=Homo sapiens GN=MUC19

Supplementary Table 2: Residual distance Between residues at Wild Type and Mutated state for SET8

Residue positions	Type	Distance between residues (in Å)
**86-158**	Wild Type	48.4
**86-158**	Mutant	43.4
**86-159**	Wild Type	51.4
**86-159**	Mutant	41.2
**86-162**	Wild Type	50.4
**86-162**	Mutant	38.2
**86-164**	Wild Type	45.8
**86-164**	Mutant	43.2
**86-168**	Wild Type	36.6
**86-168**	Mutant	50.7
**86-169**	Wild Type	39.4
**86-169**	Mutant	50.6
**86-170**	Wild Type	42.4
**86-170**	Mutant	49.5
**86-174**	Wild Type	40
**86-174**	Mutant	54.7
**158-168**	Wild Type	13.3
**158-168**	Mutant	19
**158-169**	Wild Type	10.6
**158-169**	Mutant	20.4
**158-170**	Wild Type	9.3
**158-170**	Mutant	17.1
**158-174**	Wild Type	17.4
**158-174**	Mutant	16.2

**162-168**	Wild Type	17.4
**162-168**	Mutant	15.8
**162-169**	Wild Type	15.5
**162-169**	Mutant	17
**162-170**	Wild Type	14.6
**162-170**	Mutant	14.7
**162-174**	Wild Type	23.2
**162-174**	Mutant	17.7

Supplementary File 1: All primer sequences mentioned in the "Methods" section

Primer used for mutagenesis:
SET8-K86-F
/5Phos/GCCGGAATCTACAGGAAACGAGAAGAGAAAAG
SET8-K86A-R
/5Phos/TAATGGTGCCCCCTGGCATTTGACTTCGTGATG
SET8-157-R
/5Phos/CAGGGCTTGCTTGGCGATGGCTGCATTGGT
SET8-K158/9/162/4A-F
/5Phos/GCAGCGCCCATCGCGGGCGCACAGGCC
SET8-165aa-F
/5Phos/AGGCCCCCCGAAAAAAAGCTCAAGGAAAAACG
SET8-165aa-R
/5Phos/GTTTGCCCTTGATGGGCTTTTTCAGGGCTTGC
SET8-R168K169K170A-F
/5Phos/AGGCCCCCGCAGCAGCAGCTCAAGGAAAAACG
SET8-K159A-R
/5Phos/GTTTGCCCTTGATGGGCGCTTTCAGGGCTTGC

Primer used for cloning:
GST-SET8-1-F
CGTCAGGGATCCCCGCTAGAGGCAGGAAGATGTCCAAGCCC
GST-SET8-352-R
CGTCAGGGATTCTTAATGCTTCAGCCACGGGTGGGCTTC
GST-SET8-81-F
CGTCAGGGATCCCCGAAGTCAAATGCCAGGGGAAACCATTAGCC
GST-SET8-230-R
CGTCAGGAATTCCACACCCCTGCCTTTGCCATCGATGAG
GST-SET8-80-R
AGTCGTGAATTCTTCGTGATGTGTAACTGAGTTCTCTTCCTG
GST-SET8-90-R
CGTCAGGAATTCGATTCCGGCTAATGGTTTCCCCTG
GST-SET8-98-F
CGTCAGGGATCCCCAGAAATGCTGGGAACGCAGTACGG
GST-SET8-108-F
CGTCAGGGATCCCCATGAAGTCCGAGGAACAGAAGATC
GST-SET8-118-F
CGTCAGGGATCCCCGCCAGGAAAGGTCCCCTGGTACCT
GST-SET8-155-R
CGTCAGGGAATTCTTGCTTGGCGATGGCTGCATTGGT
GST-SET8-157-F
CGTCAGGAATCCCCAAAAAGCCCATCAAGGGC
GST-SET8-170-F

CGTCAGGAATCCCCGCTCAAGGAAAAACGCAAC
GST-SET8-180-F
CGTCAGGAATCCCCCTTACGGATTTCTACCCT
GST-SET8-190-F
CGTCAGGGATCCCCTCCAGGAAGAGCAAAGCC
GST-SET8-200-F
CGTCAGGGATCCCCGAAGAAAGGAAAAGAATAGATGA
GFP-SET8-1-F
CGTCAGGAATTCGCTAGAGGCAGGAAGATGTCCAAGCC
GFP-SET8-352-R
CGTCAGGGATCCTTAATGCTTCAGCCACGGGT
GST-PARP1-1-F
CATCATGTCGACCCGCGGAGTCTTCGGATAAGCTCTATCGA
GST-PARP1-372-R
CATCATGCGGCCGCCACAGCAGCAGGAGCCGA
GST-PARP1-373-F
CATCATGTCGACCCGAAACCAGCGCCTCCGTG
GST-PARP1-532-R
CATCATGCGGCCGCGAATCAGGATCCACAGCTGCTCC
GST-PARP1-533-F
CATCATGTCGACCCAAAGGAGGAGCAGCTGTGGAT
GST-PARP1-1014-R
CATCATGCGGCCGCTTACCACAGGGAGGTCTTAAAATTGAATTTCAG
3XFLAG-PARP1-1-F
CATCATGCGGCCGCGGCGGAGTCTTCGGATAAGCT
3XFLAG-PARP1-1014-R
CATCATAGATCTTTACCACAGGGAGGTCTTAAAATTGAA
