# Supplementary material for: Poly ADP-ribosylation of SET8 leads to aberrant H4K20 methylation in mammalian nuclear genome
Source: Commun Biol. 2022 Nov 25;5:1292. doi: 10.1038/s42003-022-04241-8 (PMC9700808; doi:10.1038/s42003-022-04241-8)
Supplement: Supplementary file 3 — Description of Additional Supplementary Files [file 42003_2022_4241_MOESM3_ESM.pdf]

Description of Additional Supplementary Files

File name: Supplementary Data 1
Description: The Source data for Supplementary Figure 13 and Figure 15

File name: Supplementary Data 2
Description: The Source data for Figure 5a-b

File name: Supplementary Data 3
Description: The Source data for Figure 3c

File name: Supplementary Data 4
Description: The Source data for Supplementary Figure 1a

File name: Supplementary Data 5
Description: The Source data for Figure 4b and Supplementary Figure 17b

File name: Supplementary Data 6
Description: The Source data for Supplementary Figure 12 and Supplementary Figure 14

File name: Supplementary Data 7
Description: The Source data for Supplementary Figure 16

File name: Supplementary Data 8
Description: The Source data for Figure 4c

File name: Supplementary Data 9
Description: RNA seq of genes post PARP1 knock down
