# Supplementary material for: Poly ADP-ribosylation of SET8 leads to aberrant H4K20 methylation in mammalian nuclear genome
Source: Commun Biol. 2022 Nov 25;5:1292. doi: 10.1038/s42003-022-04241-8 (PMC9700808; doi:10.1038/s42003-022-04241-8)
Supplement: Supplementary file 13 — Reporting Summary [file 42003_2022_4241_MOESM13_ESM.pdf]

nature portfolio

Corresponding author(s): Sriharsa Pradhan

Last updated by author(s): Sep 29, 2022

Reporting Summary

Nature Portfolio wishes to improve the reproducibility of the work that we publish. This form provides structure for consistency and transparency in reporting. For further information on Nature Portfolio policies, see our Editorial Policies and the Editorial Policy Checklist.

Statistics

For all statistical analyses, confirm that the following items are present in the figure legend, table legend, main text, or Methods section.

n/a	Confirmed	
☐	☒	The exact sample size (*n*) for each experimental group/condition, given as a discrete number and unit of measurement
☐	☒	A statement on whether measurements were taken from distinct samples or whether the same sample was measured repeatedly
☐	☒	The statistical test(s) used AND whether they are one- or two-sided<br>*Only common tests should be described solely by name; describe more complex techniques in the Methods section.*
☐	☒	A description of all covariates tested
☐	☒	A description of any assumptions or corrections, such as tests of normality and adjustment for multiple comparisons
☐	☒	A full description of the statistical parameters including central tendency (e.g. means) or other basic estimates (e.g. regression coefficient) AND variation (e.g. standard deviation) or associated estimates of uncertainty (e.g. confidence intervals)
☐	☒	For null hypothesis testing, the test statistic (e.g. *F*, *t*, *r*) with confidence intervals, effect sizes, degrees of freedom and *P* value noted<br>*Give P values as exact values whenever suitable.*
☒	☐	For Bayesian analysis, information on the choice of priors and Markov chain Monte Carlo settings
☐	☒	For hierarchical and complex designs, identification of the appropriate level for tests and full reporting of outcomes
☐	☒	Estimates of effect sizes (e.g. Cohen's *d*, Pearson's *r*), indicating how they were calculated

Our web collection on statistics for biologists contains articles on many of the points above.

Software and code

Policy information about availability of computer code

Data collection	Not applicable
Data analysis	The raw fastq sequences were trimmed using Trim Galore (http://www.bioinformatics.babraham.ac.uk/projects/trim_galore/) to remove the adapters and low-quality sequences. Trimmed reads were mapped to the human reference assembly hg38 using Bowtie2 (45). Aligned reads in bam format were filtered for duplicates and low-quality alignments using Picard (http://broadinstitute.github.io/picard/) and samtools (46). The aligned bam files of technical replicates were merged using Sambamba (47). H4K20me1 enriched regions were identified by calling broad peaks (target over input) using MACS2 (48) where the parameter broad-cutoff was set to 0.025 for more robustness . Signal tracks were generated using deeptools bamCoverage (49) with the parameters, -normalizeUsing RPKM -of bigwig -e. Spearman correalation analysis was performed using deeptools plotCorrelation (49) function. H4K20me1 peaks were annotated using HOMER (50) annotatePeaks.pl. Repeats elements were annotated using HOMER (50). H4K20me1 profile in the gene regions for PARP1 from Hela cells for bothe the conditions was computed with the deeptools computeMatrix and plotProfile (49) functions. Peak length was calculated for all the conditions to estimate the gain and loss of H4K20me1 after PARP1 knock down. Then the enrichment scores were summarized into a data matrix in R (51) and a heatmap was then created using heatmap.2 function to represent condition-specific enrichment of TF binding motifs near the H4K20me1 peaks. Genomic regions were visualized using Integrative genomic viewer (IGV) (53).

For manuscripts utilizing custom algorithms or software that are central to the research but not yet described in published literature, software must be made available to editors and reviewers. We strongly encourage code deposition in a community repository (e.g. GitHub). See the Nature Portfolio guidelines for submitting code & software for further information.

Data

Policy information about availability of data

All manuscripts must include a data availability statement. This statement should provide the following information, where applicable:

- Accession codes, unique identifiers, or web links for publicly available datasets
- A description of any restrictions on data availability
- For clinical datasets or third party data, please ensure that the statement adheres to our policy

ChIP-seq and NicE-seq data performed in this study are available in NCBI Gene Expression Omnibus (GEO) under the accession GSE188744

Human research participants

Policy information about studies involving human research participants and Sex and Gender in Research.

Reporting on sex and gender	Not applicable
Population characteristics	Not applicable
Recruitment	Not applicable
Ethics oversight	Not applicable

Note that full information on the approval of the study protocol must also be provided in the manuscript.

Field-specific reporting

Please select the one below that is the best fit for your research. If you are not sure, read the appropriate sections before making your selection.

☒ Life sciences ☐ Behavioural & social sciences ☐ Ecological, evolutionary & environmental sciences

For a reference copy of the document with all sections, see nature.com/documents/nr-reporting-summary-flat.pdf

Life sciences study design

All studies must disclose on these points even when the disclosure is negative.

Sample size	Not applicable
Data exclusions	Not applicable
Replication	Replication was successful
Randomization	*Describe how samples/organisms/participants were allocated into experimental groups. If allocation was not random, describe how covariates were controlled OR if this is not relevant to your study, explain why.*
Blinding	*Describe whether the investigators were blinded to group allocation during data collection and/or analysis. If blinding was not possible, describe why OR explain why blinding was not relevant to your study.*

Reporting for specific materials, systems and methods

We require information from authors about some types of materials, experimental systems and methods used in many studies. Here, indicate whether each material, system or method listed is relevant to your study. If you are not sure if a list item applies to your research, read the appropriate section before selecting a response.

Materials & experimental systems

n/a	Involved in the study
☐	☒ Antibodies
☐	☒ Eukaryotic cell lines
☒	☐ Palaeontology and archaeology
☒	☐ Animals and other organisms
☒	☐ Clinical data
☒	☐ Dual use research of concern

Methods

n/a	Involved in the study
☐	☒ ChIP-seq
☒	☐ Flow cytometry
☒	☐ MRI-based neuroimaging

Antibodies

Antibodies used

Antibodies against SET8 were obtained from Cell Signaling Technology (# 2996), ABCAM (# ab3798) and Santa Cruz Biotechnology (# sc-515433). Anti-PARP1 (# 9532) and anti-ADPribose antibody (# 83732S) were purchased from Cell Signaling Technology. Anti-H4K20me1 (# MA5-18067), anti-histone H4K20me2 (# 9759) and anti-H4K20me3 (# 5737) were obtained from Thermo Fisher Scientific and Cell Signaling Technology respectively and used at 1/1000 dilution. Anti-β-actin (# 4970S) and anti-GFP (# 11814460001) antibodies were obtained from Cell Signaling Technology and Millipore-Sigma respectively and used at a 1:5000 dilution. HCT116 or HeLa cells using anti-PARP1 antibody (Cell Signaling Technology # 9532), anti-SET8 antibody (Santa Cruz Biotechnology # sc-515433) or 5 µg of rabbit IgG as a control antibody (Santa Cruz Biotechnology # sc-2027). Immuno-Precipitation reactions were blotted using anti-PARP1 antibody (Millipore-Sigma # HPA045168) and rabbit anti-SET8 antibody (Cell Signaling Technology # 2996S). For immunoprecipitation of SET8 from synchronized HeLa cells, 200 µg of chromatin cell lysate was incubated with 5 µg of anti-SET8 (sc-515433, Santa Cruz Biotechnology).

Validation

Describe the validation of each primary antibody for the species and application, noting any validation statements on the manufacturer's website, relevant citations, antibody profiles in online databases, or data provided in the manuscript.

Eukaryotic cell lines

Policy information about cell lines and Sex and Gender in Research

Cell line source(s)	ATCC (HeLa, HCT116 and COS-7 cells)
Authentication	Not Applicable
Mycoplasma contamination	Mycoplasma free
Commonly misidentified lines (See ICLAC register)	Not applicable

ChIP-seq

Data deposition

☒ Confirm that both raw and final processed data have been deposited in a public database such as GEO.

☒ Confirm that you have deposited or provided access to graph files (e.g. BED files) for the called peaks.

Data access links *May remain private before publication.*	NCBI Gene Expression Omnibus (GEO) under the accession GSE188744
Files in database submission	SiPARP1_H4K20me3 (Rep1/2) SiPARP1_H4K20me1 (Rep1/2) SiGFP_H4K20me3 (Rep1/2) SiGFP_H4K20me1 (Rep1/2) SiPARP1_Input (Rep1/2) SiGFP_Input (Rep1/2) SiPARP1_RNAseq (Rep1/2) SiGFP_RNAseq (Rep1/2)
Genome browser session (e.g. UCSC)	Not Applicable

Methodology

Replicates	Two biological replicates in all western blot down cytometry analysis
Sequencing depth	siGFP_H420me1= 27798385 (reads), siGPF_H4K20me3=35593041(reads), siPARP1_H4K20me1= 23044722, siPARP1_H4K20me3=25374497<br>All the data are paired end data.
Antibodies	ChIP-seq of H4K20me1 or H4K20me3 was performed using antibody from Thermo Fisher Scientific # MA5-18067, Abcam # ab9053 respectively.
Peak calling parameters	--broad with --broad-cutoff=0.025

Data quality

Describe the methods used to ensure data quality in full detail, including how many peaks are at FDR 5% and above 5-fold enrichment.

Software

The raw fastq sequences were trimmed using Trim Galore (http://www.bioinformatics.babraham.ac.uk/projects/trim_galore/) to remove the adapters and low-quality sequences. Trimmed reads were mapped to the human reference assembly hg38 using Bowtie2 (45). Aligned reads in bam format were filtered for duplicates and low-quality alignments using Picard (http://broadinstitute.github.io/picard/) and samtools (46). The aligned bam files of technical replicates were merged using Sambamba (47). H4K20me1 enriched regions were identified by calling broad peaks (target over input) using MACS2 (48) where the parameter broad-cutoff was set to 0.025 for more robustness . Signal tracks were generated using deeptools bamCoverage (49) with the parameters, -normalizeUsing RPKM -of bigwig -e. Spearman correalation analysis was performed using deeptools plotCorrelation (49) function. H4K20me1 peaks were annotated using HOMER (50) annotatePeaks.pl. Repeats elements were annotated using HOMER (50). H4K20me1 profile in the gene regions for PARP1 from Hela cells for bothe the conditions was computed with the deeptools computeMatrix and plotProfile (49) functions. Peak length was calculated for all the conditions to estimate the gain and loss of H4K20me1 after PARP1 knock down. Then the enrichment scores were summarized into a data matrix in R (51) and a heatmap was then created using heatmap.2 function to represent condition-specific enrichment of TF binding motifs near the H4K20me1 peaks. Genomic regions were visualized using Integrative genomic viewer (IGV) (53).
